# The impact of Ramadan intermittent fasting on anthropometric measurements and body composition: Evidence from LORANS study and a meta-analysis

**DOI:** 10.3389/fnut.2023.1082217

**Published:** 2023-01-17

**Authors:** Rami Al-Jafar, Nisa Sri Wahyuni, Karim Belhaj, Mohammad Hamed Ersi, Zahra Boroghani, Amer Alreshidi, Zahra Alkhalaf, Paul Elliott, Konstantinos K. Tsilidis, Abbas Dehghan

**Affiliations:** ^1^Department of Epidemiology and Biostatistics, School of Public Health, Imperial College London, London, United Kingdom; ^2^Department of Data Services, Lean Business Services, Riyadh, Saudi Arabia; ^3^Faculty of Medicine, Hormozgan University of Medical Sciences, Bandar Abbas, Iran; ^4^Clinical Research Development of Shahid Mohammadi Hospital, Hormozgan University of Medical Sciences, Bandar Abbas, Iran; ^5^Pharmaceutical Care Department, Hail General Hospital, Hail Health Cluster, Ministry of Health, Hail, Saudi Arabia; ^6^Dammam Medical Complex, Medical and Clinical Affairs, Dammam, Saudi Arabia; ^7^Dementia Research Institute at Imperial College London, London, United Kingdom; ^8^National Institute for Health Research Imperial College Biomedical Research Centre, Imperial College London, London, United Kingdom; ^9^Department of Hygiene and Epidemiology, University of Ioannina School of Medicine, Ioannina, Greece; ^10^MRC-PHE Centre for Environment and Health, School of Public Health, Imperial College London, London, United Kingdom

**Keywords:** anthropometry, weight, waist circumference, body mass index, fat, muscle mass, systematic review

## Abstract

**Background:**

Although the effect of Ramadan intermittent fasting (RIF) on anthropometry and body composition has been questioned, none of the previous studies tried to explain the reported changes in these parameters. Also, systematic reviews that investigated the topic were limited to healthy individuals or a specific disease group.

**Methods:**

The London Ramadan Study (LORANS) is an observational study on health effects of RIF. We measured weight, waist circumference (WC), hip circumference (HC), body mass index (BMI), waist-to-hip ratio (WHR), basal metabolic rate (BMR), fat percentage (FP), free-fat mass (FFM), extremities predicted muscle mass, total body water (TBW), trunk FM, trunk FFM and trunk predicted muscle mass before and immediately after Ramadan. Using mixed-effects regression models, we investigated the effect of RIF with adjustment for potential confounders. We also conducted a meta-analysis of the results of LORANS with other studies that investigated the effect of RIF on anthropometry and body composition. The review protocol is registered with PROSPERO registry (CRD42020186532).

**Results:**

We recruited 146 participants (Mean ± SD age = 43.3 ± 15 years). Immediately after Ramadan, compared with before Ramadan, the mean difference was−1.6 kg (*P*<0.01) in weight,−1.95cm (*P*<0.01) in WC,−2.86cm (*P* <0.01) in HC, −0.60 kg/m^2^ (*P* < 0.01) in BMI and −1.24 kg (*P* < 0.01) in FM. In the systematic review and meta-analysis, after screening 2,150 titles and abstracts, 66 studies comprising 7,611 participants were included. In the general population, RIF was followed by a reduction of 1.12 Kg in body weight (−1.89– −0.36, I^2^ = 0), 0.74 kg/m^2^ reduction in BMI (−0.96– −0.53, I^2^ = 0), 1.54cm reduction in WC (−2.37– −0.71, I^2^ = 0) and 1.76cm reduction in HC (−2.69– −0.83, I^2^ = 0). The effect of fasting on anthropometric and body composition parameters starts to manifest in the second week of Ramadan and starts to diminish 3 weeks after Ramadan.

**Conclusion:**

RIF is associated with a reduction in body weight, BMI, WC, HC, FM, FP and TBW. Most of these reductions are partially attributed to reduced FM and TBW. The reductions in these parameters appear to reverse after Ramadan.

## Introduction

In the last few decades, more fasting regimens, usually referred to as intermittent fasting, have emerged as a non-pharmaceutical approach in integrative medicine and a method to reduce and control weight to improve health (1, 2). There are different types of intermittent fasting, but the most common ones are alternate-day fasting, time-restricted feeding, twice-a-week method and 24 h fasting (2). Ramadan intermittent fasting (RIF) is a model of time-restricted feeding. Every year, hundreds of millions of Muslims observe RIF. During Ramadan, Muslims fast from dawn to sunset and adopt a different lifestyle that could affect their health. Inevitably, this dramatic change in their dietary habits and lifestyle will be reflected in their anthropometric measurements and body composition, which are tightly connected to their health status. For instance, body weight and shape influence metabolism and insulin sensitivity (3, 4). Most studies that have investigated the effect of RIF on anthropometric and body composition parameters concluded that RIF is associated with lower weight, body mass index (BMI), fat mass (FM), fat percentage (FP), waist circumference (WC) and hip circumference (HC) (5–9). However, a few studies showed no changes or even an increase in these parameters (10–17). Moreover, none of the studies showing reductions in these parameters considered potential confounders that may have affected their findings or explained the observed changes (13, 14, 18–20) and none targeted the general population.

Furthermore, to date, seven meta-analyses have been done on the effect of RamadanRIF on body weight (21–24), BMI (24, 25), WC (26), FP (27), FM (24, 27), and fat-free mass (FFM) (24, 27). Still, none has done a meta-analysis on the effect on HC, muscle mass, total body water (TBW), and waist-to-hip ratio (WHR). The studies on BMI and FFM reported heterogeneous results. Also, except for one review which focused on type 2 diabetes patients (25), all others were limited to healthy individuals; therefore, the findings of these studies are not generalisable to the Muslim population (healthy and unhealthy). Using time of follow-up visits as a subgrouping variable, two reviews (23, 27) conducted a meta-analysis on the effect estimates at two time-points of follow-up (last week of Ramadan–a week after Ramadan; more than 2 weeks after Ramadan), but the other reviews observed the effect at only one time-point of follow-up. Conducting analyses at various time points of follow-up is required to know when the effects start to take place during Ramadan and whether they persist or fade away after Ramadan.

Therefore, to bridge these knowledge gaps, we conducted the London Ramadan Study (*LORANS*), which is a community-based study targeting the general population, in which we collected data on anthropometry and body composition of individuals as well as potential confounders to apply appropriate adjustments and explain the changes in the investigated parameters. And we conducted a systematic review and meta-analysis (including subgroup analysis based on time-points of follow-up) to investigate the effect of RIF on BMI, weight, FM, muscle mass, TBW, WC, HC, and WHR.

## Methods

### LORANS

*LORANS* is an observational study among the general population of Muslims in London during Ramadan 2019 (25 April−16 June 2019). We contacted six large mosques in London and provided them with information about the study. Out of the six, five mosques (Beitulfutuh Mosque, Finsbury Park Mosque, West London Mosque, Almanaar Mosque, and Madina Mosque) agreed to support the study and dedicate a room for recruitment and data collection. Before data collection, from 19 to 24 April, we launched a temporary website (https://fastingresearch.co.uk/) that provided information about the study and its aims and had a booking link enabling interested individuals to book their visits in one of the data collection settings. Further, we visited the five mosques to invite individuals who attended prayer sessions to take part in LORANS and distributed flyers about the study. And we asked mosque leaders to arrange for an announcement about our study right after a Friday midday prayer a couple of weeks before our data collection period.

We set up clinics in the five mosques where participants were recruited and data were collected. Within 10 days before Ramadan, we collected data on 146 participants from the five mosques. Eighty-five participants came for a second measurements visit which was 8–12 days after Ramadan. A comparison between participants who completed the study and those who only attended the first visit showed that the two groups were largely similar except for age and smoking status (Supplementary material 1).

We included all individuals 18 years old and over who were planning to fast for at least 20 days of Ramadan. Pregnant women and those who were not going to be available after Ramadan were excluded.

The measurements were collected from participants before Ramadan (from 26 to 30 April from 1 to 7 pm) and after Ramadan (from 10 to 14 June from 1 to 7 pm). We followed the Airwave study protocol (28) in data collection. We measured height, weight, WC and HC using a stadiometer (Leicester Height Measure), a weighing scale (Marsden digital weighing scale) and a body tape measure. Measurements were reported to the nearest 0.1 kg and 0.01. Each anthropometric measurement was repeated twice, and the average of the two measurements was recorded. Weight was measured without heavy clothes/items and shoes. WC was measured by wrapping the tape measure at the top of the hip bones or the narrowest area to the umbilicus if it was difficult to locate the hip bones. The HC was measured by wrapping the tape measure around the broadest area of the hips. Participants' body composition was measured using a bioelectrical impedance analyser (BIA) (model: Tanita BC-418) with substracting 2 kg from weight measured by the BIA to correct for clothes. For accuracy and safety, before using the BIA, we asked participants whether they have metal implants or pacemakers. Participants' body composition was measured using a multi-frequency segmental bioelectrical impedance analyser (BIA) (model: Tanita BC-418). This BIA model is built to last for around 300,000 measurements without the need of calibration and has eight tactile electrodes for hands and feet. The BIA estimates basal metabolic rate (BMR), FP, FM, FFM, and TBW. The BIA also estimates FP, FM, FFM and predicted muscle mass (PMM) in the trunk and each limb using prediction equations that involve height, weight, age and sex of the participant to calculate body composition parameters (29). It uses inbuilt equations derived from previously conducted regression analyses with Dual Energy X-ray Absorptiometry (DEXA). BIA has been validated in studies using DEXA and magnetic resonance imaging (30, 31). Participants were asked to take off heavy clothes and shoes before taking their measurements or using the BIA. Also, we wiped tactile electrodes with disinfectant Wipes after each use. We calculated BMI by dividing weight (kg) by height in meters squared. We took the average of the four extremities' predicted muscle masses to estimate extremities PMM.

All participants submitted an online questionnaire about their lifestyle and socioeconomic status. Also, 55 participants filled out a 3-day food diary before and during Ramadan. The 3 days consisted of 2 weekdays and 1 weekend day to capture changes in diet during weekends. The food diary included instuctions about how to fill it out and examples for the participants. Nutritics (nutrition software) was used for food diary analysis (32).

The ethics committee at Imperial College London approved the study (reference: 19IC5138, dated 17/4/2019), and all participants gave informed consent before taking part.

### Systematic review

We conducted a systematic review following the PRISMA guidelines (Supplementary material 2) and registered the review protocol under the PROSPERO registry of CRD42020186532.

#### Eligibility criteria

We sought published and unpublished studies that examined the effect of RIF on anthropometric measurements and/or body composition. We considered studies that measured any anthropometric or a body composition measure before and during/after Ramadan. We only included studies that reported measurements as means and SDs to obtain the pooled effect. We excluded reviews, studies that involved any other intervention besides RIF, studies on pregnant women and studies of athletes.

#### Information sources, search and study selection

We searched three databases: PubMed, Embase and Scopus from inception until 6 May 2022, seeking all relevant published studies without language restrictions. We used this search approach “Ramadan” or “Ramadan fasting” or “Ramadan intermittent fasting” and (“anthropometr^*^” or “weight” or “BMI” or “fat” or “body composition” or “waist circumference” or “obes^*^” or “body mass index” or “hip circumference”).

Each of the following procedures was carried out twice and in parallel by two independent reviewers (divided among RA, NW, KB, ME, ZB, ZA, and AA). We screened all titles and abstracts of all potential studies; then we assessed the full-texts of studies that were deemed eligible. We resolved disagreements by consensus.

#### Data collection process

RA, NW, ME and ZB extracted all relevant data from enrolled studies in a spreadsheet. Extracted data included: author, year, study country, journal, sample size, population type, tools used for measurements, means and SDs of anthropometric and body composition measurements including weight, height, BMI, WC, HC, basal metabolic rate (BMR), FM, muscle mass, FFM, TBW and WHR in all time-points; information on adjustments (if any).

#### Risk of bias

We modified the Newcastle-Ottawa Quality Assessment Scale of cohort studies (33) to make a version that could be applied in this review. The scale (Supplementary material 3) is based on selection, comparability and outcome. Based on the quality score, we classified studies as either low quality (0–4), satisfactory (5, 6), good (7, 8) or very good (9). RA, NW, ME and ZB assessed the quality of all selected studies independently in parallel and resolved disagreements by consensus. Supplementary material 4 shows the quality scores of selected studies.

#### Estimating fasting time length

We adopted the same method developed by Faris et al. (26), which basically relies on “timeanddate” (https://www.timeanddate.com/sun/@8469718) to estimate the day length (from sunrise and sunset). Since Muslims start fasting around 80 min before sunrise (Al-Fajr prayer call), 80 min should be added to the day length to estimate the fasting time.

### Statistical analysis

#### LORANS

Using multiple imputations with (“mice”) package in R (Windows 3.6.1), we imputed the second measurements of weight, WC, HC, BMR, FM, FP, FFM, TBW, extremities PMM, trunk FM, trunk FFM and trunk PMM for the 61 participants who didn't attend the second visit. Afterwards, we calculated BMI and WHR based on the imputed values.

We used R (“lme4” package) to apply linear mixed-effects model regression to estimate the impact of RIF on anthropometric measurements and body composition. The linear mixed-effects model allowed us to correct for potential confounders, including both fixed and random factors. The dependent variables were the anthropometric and body composition parameters. The independent variables were age, sex, number of fasting days during Ramadan, day of the second measurement, and location (mosque). Also, we adjusted for energy intake, extremities PMM, fat mass and TBW to explore to what extent these parameters contribute to the changes in the other dependent variables. We ran the analysis using five models. In the first model, we adjusted for age, sex, number of fasting days, second visit day and location. Other models were further adjusted for energy intake (model 2); extremities PMM (model 3); FM (model 4); and TBW (model 5). These adjustments were applied to investigate the effects of potential confounders (which were measured before and after Ramadan) and to explain the observed changes in anthropometric measurements and body composition. Moreover, we grouped the participants based on their baseline BMI (normal weight = 18.5– <25; overweight = 25.0– <30; obesity = ≥30 kg/m^2^) and retested the changes of anthropometric and body composition indices (using the first model discussed above). When the correlation coefficient was >0.6 between the outcome and potential confounders, we did not apply the model to avoid collinearity. We considered the result to be statistically significant if the *p* < 0.05.

#### Meta-analysis

We used R (Windows 3.6.1, “meta” package) to run an inverse variance weighted fixed-effects model to estimate the pooled effect (difference in means after/before Ramadan from included studies) of RIF on anthropometric and body composition measures. Also, we estimated the consistency of included studies by a test of heterogeneity (I^2^). We grouped studies based on the timing of their follow-up visits. To collate studies with similar follow-up, we grouped studies into four time-points of follow-up: middle of Ramadan (2^nd^/3^rd^ week of Ramadan), end of Ramadan (4^th^ week of Ramadan), immediately after Ramadan (within 2 weeks after Ramadan) and long after Ramadan (three to 6 weeks after Ramadan). Meta-analysis was separately done for each measured parameter which were BMI, weight, FP, FM, WC, HC, WHR, TBW and muscle mass. If a study measured a parameter at two different time points (e.g., 4^th^ week of Ramadan and long after Ramadan), we included it in both meta-analyses. This approach led to having 25 meta-analyses with three studies being the smallest number of studies in a meta-analysis. The pooled effect with a *p* < 0.05 was deemed statistically significant.

The potential presence of the small-study effect was assessed qualitatively (visualizing funnel plots) and quantitively (using Egger's regression tests) (34). If Egger's test had a *p* < 0.1, we applied the trim and fill method to address the asymmetry. Then, we re-estimated the effect under this correction and compared it to the original effect.

## Results

### LORANS

**Figure 1:**
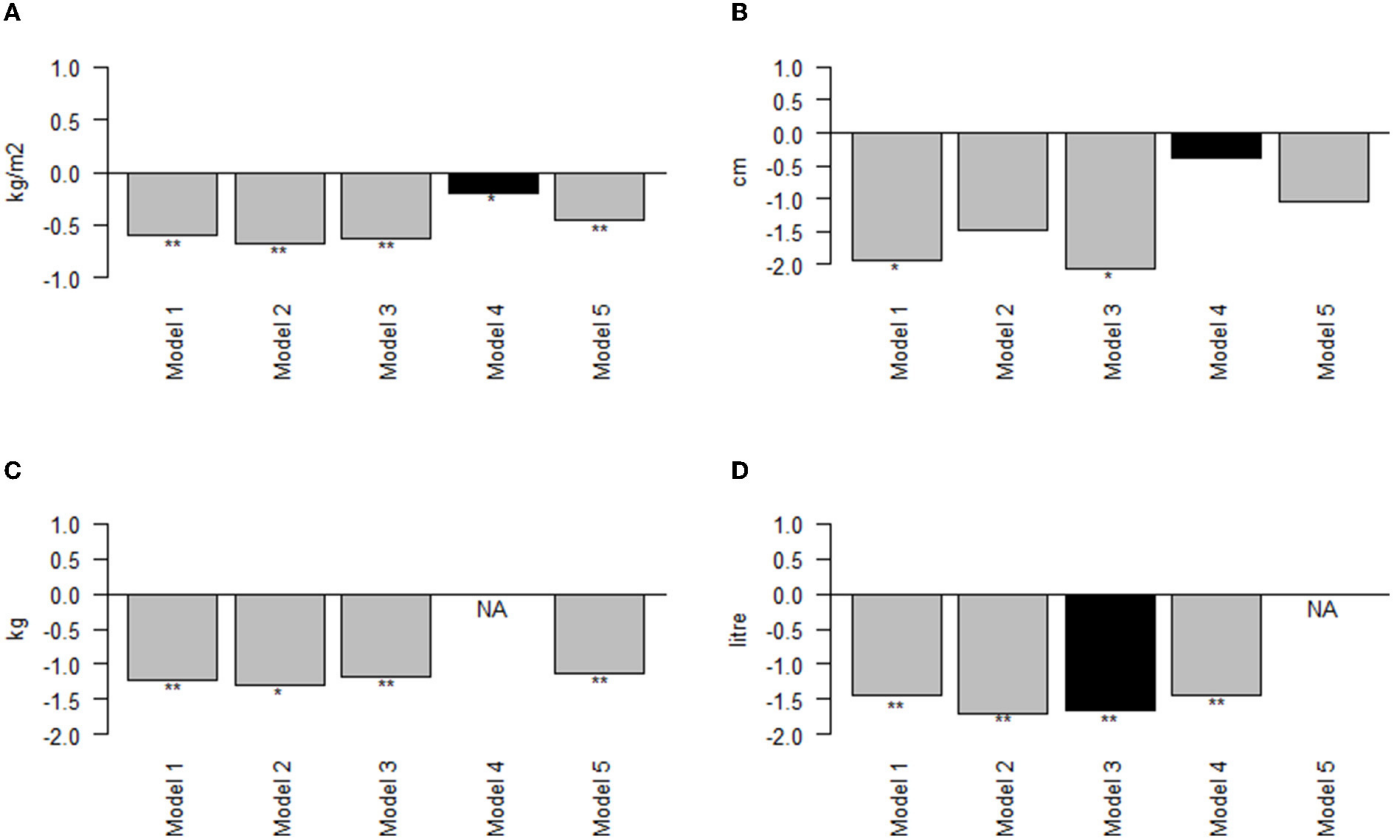
Effect of adjustments on mean difference in BMI, waist circumference, fat mass and total body water from *LORANS*. **(A)** Mean difference in BMI; **(B)** Mean difference in waist circumference; **(C)** Mean difference in fat mass; **(D)** Mean difference in total body water; ^*^: *P* < 0.05; ^**^: *P* < 0.001; NA, the model adjusts for the outcome; Black bar, the outcome is highly correlated (r > 0.6) with the variable adjusted for.

Supplementary material 5 shows the participants' baseline characteristics. Out of the 146 participants, 75 (51.4%) participants were male and mean ± SD age was 43.3 ± 15 years. During Ramadan, total energy intake was 1,507 kcal compared to 1,503 kcal before Ramadan (*P* = 0.940). Similarly, no significant changes were reported in intakes of carbohydrates (16.9 g, 0.3–34, *P* = 0.214), protein (−7.4, −16.1–1.3, *P* = 0.110) or fat (−3.8 g, −10.4–3, *P* = 0.274) during Ramadan. After Ramadan, there was a significant reduction of 1.6 kg (*P* < 0.01) in body weight, 1.95 cm (*P* < 0.01) in WC, 2.86 cm (*P* < 0.01) in HC, 0.60 kg/m^2^ (*P* < 0.01) in BMI, 1.05% (*P* < 0.01) in FP, 1.24 kg (*P* < 0.01) in FM and 1.45 liter (*P* < 0.01) in TBW (Figure 1). We also observed non-significant decreases in FFM (−0.21 kg), trunk FP (−0.85 kg) and trunk FM (−0.53 kg). The change in extremities' PMM was non-significant and negligible. BMR decreased by −12.8 Kcal (*P* < 0.05) after Ramadan. Further, adjustment for energy intake only diminished the decrease in WC (from −1.95 to −1.49 cm), and trunk FP (from −0.85 to −0.73). None of the observed differences was influenced by adjusting for extremities PMM in the third model. Almost all mean differences were influenced by the fourth and fifth models. After adjustment for FM, the reductions in weight and trunk FFM became non-significant. Also, the reduction in WC became non-significant when adjusted for TBW in the fifth model (Supplementary material 6).

When we divided participants into groups based on baseline BMI, we noticed that the weight and BMI reductions were non-significant in individuals with obesity. And HC and TBW reductions were non-significant in individuals with normal weight (Supplementary material 7).

### Systematic review

**Figure 2:**
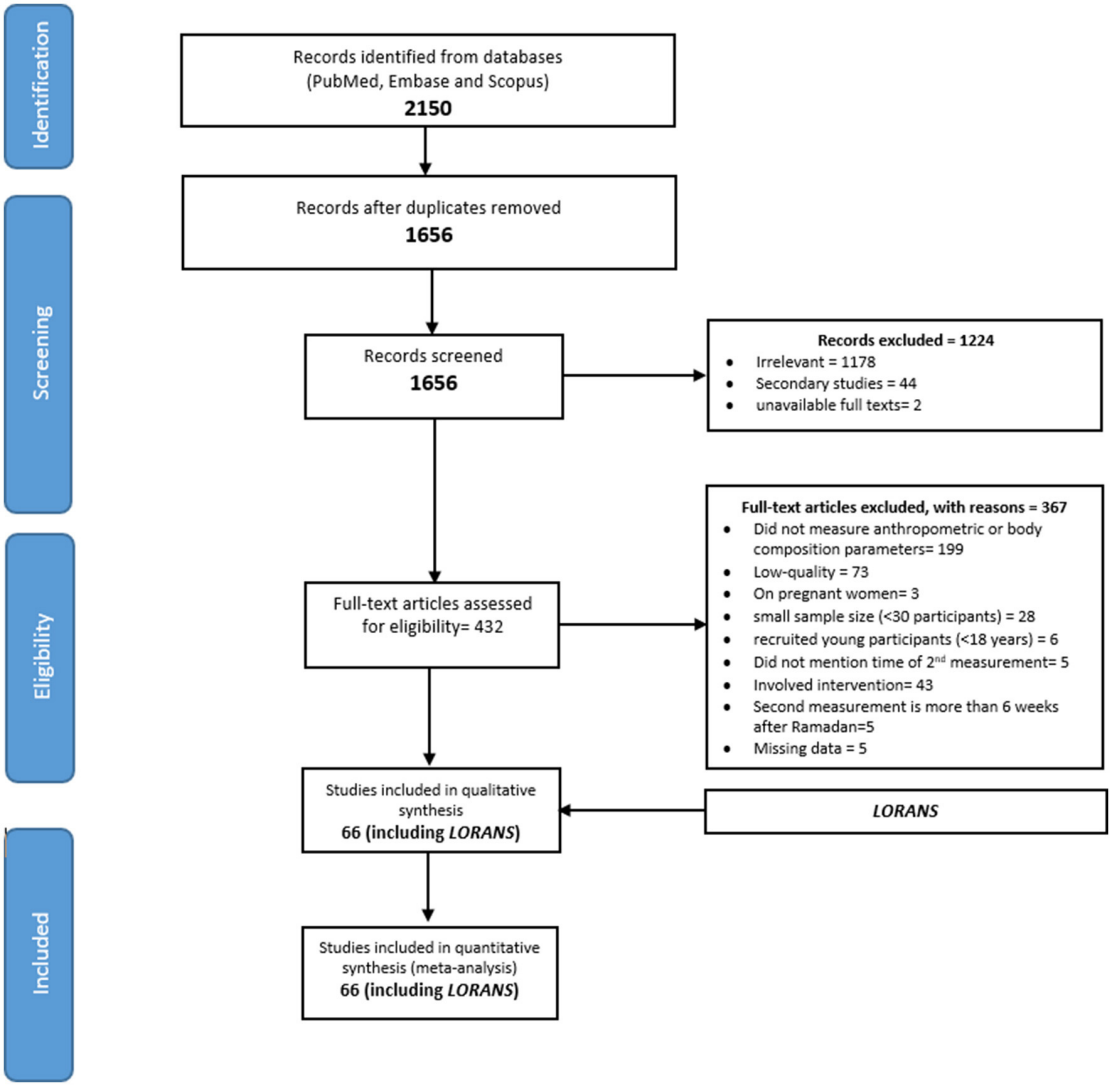
Flowchart of study selection.

After screening 2,150 titles /abstracts and reading 432 full texts, we identified 139 studies (Figure 2). Of these, 73 studies had low-quality scores (quality score average = 3.85 out of 9) and were not included in the review. As a result, we included 66 studies (including *LORANS*) with a total of 7,496 participants in the meta-analysis. Of these, 55 studies were classified as satisfactory quality with moderate risk of bias and 10 studies as good quality with low risk of bias (Supplementary material 4).

#### Study characteristics

The included studies were from 20 countries and most commonly (15 studies, 22.7%) were conducted in Iran (Table 1). The majority of the studies targeted healthy individuals (21 studies). Others included type 2 diabetes patients (21 studies), overweight/obese individuals (four studies), chronic kidney disease (CKD) patients (five studies), and individuals with metabolic syndrome (three studies). *LORANS* was the only community-based study, targeting the general population. Weight was the most commonly investigated anthropometric index (54 studies) followed by BMI (50 studies), WC (33 studies), FP (13 studies), HC (13 studies), WHR (12 studies), FM (11 studies), MM (7 studies) and TBW (7 studies) (Supplementary material 8).

**Table 1:** Characteristics of studies included in the meta-analyses.

**References**	**Country**	**Subjects**	**Age (mean ±SD)**	**Males (%)**	**Average daylight hours (fasting hours)**	**Population**	Caloric intake during Ramadan^*^	**Variable**	**Before R**	**2nd or 3rd week of R**	**4th week of R**	**Immediately after R**	**Long after R**
Adanan et al. (7)	Malaysia	68	54.3 ± 2.2	55.2	13 h 35 min	CKD patients	↑	BMI	26 ± 4.20		25.80 ± 4.10		25.90 ± 4.20
								FM	23.20 ± 8.40		22.20 ± 8.80		23.10 ± 8.30
								FP	29 ± 8.50		28.80 ± 8.40		29 ± 8.40
								WC	92 ± 11.40		90.30 ± 10.70		91.50 ± 11.60
Adnan et al. (10)	Malaysia	35	54 ± 2.2	46	12 h 38 min	CKD patients	NA	Weight	65.20 ± 12.30		65.20 ± 12.50		
Ahmadinejad et al. (35)	Iran	81	22.7 ± 2.3	50.6	10 h 30 min	Healthy subjects	NA	BMI	21.20 ± 4.50		20.90 ± 2		
								Weight	62.40 ± 11.60		61.20 ± 10.80		
Al Awadi et al. (36)	NM	136	32 ± NM	55.1	Unknown	T1D patients	NA	Weight	74.80 ± 14.30				75 ± 14.40
								WC	89.30 ± 16.30				88.70 ± 15.80
Aliasghari et al. (37)	NM	42	36 ± 7	59.5	Unknown	NAFLD Patients	NA	BMI	30.09 ± 4.49			29.28 ± 4.14	
								Weight	83.65 ± 13.02			81.50 ± 12.80	
								WC	100.21 ± 11.02			99.26 ± 10.86	
								HC	107.02 ± 7.11			106.47 ± 7.09	
Akanji et al. (38)	Kuwait	64	48 ± 10.6	71.7	11 h 44 min	Hyperlipidaemic subjects	NA	Weight	82.40 ± 13.60			82.90 ± 14.80	
Alzoughool et al. (11)	Jordan	60	20.9 ± NM	28.3	15 h 25 min	Healthy subjects	NA	BMI	24.10 ± 4.49		23.90 ± 4.34		
								Weight	65.10 ± 13.94		64.70 ± 13.16		
								WC	78 ± 11.62		78.10 ± 10.84		
Bahmani (39)	Iran	191	30 ± NM	18.8	14 h 14 min	Prediabetic subjects	NA	BMI	29.44 ± 4.18			28.79 ± 4.25	
								Weight	69.30 ± 13.30			68.90 ± 11.30	
Bashier et al. (40)	NM	417	54 ± 11.6	41.5	Unknown	T2D patients	NA	Weight	83.90 ± 17				83.80 ± 16.60
Bernieh et al. (41)	NM	31	54 ± 4.2	61.3	Unknown	CKD patients	NA	Weight	76.40 ± 18		75 ± 17.60		75.70 ± 18
Bencharif et al. (42)	Algeria	72	51.2 ± 4.7	48.6	10 h 57 min	T2D patients	NA	BMI	28 ± 4.30	27.90 ± 4.30			28.60 ± 4.30
								Weight	75.40 ± 11.30	75.20 ± 11.40			77.1 ± 11.5
								FP	33.40 ± 8.60	34.60 ± 8.50			33.70 ± 8.10
								WC	94.40 ± 6.30	95 ± 6.40			96.40 ± 6.10
Bouida et al. (18)	Tunisia	98	59.1 ± 10	87.7	12 h	Individuals with high cardiovascular risk	↓	BMI	29.50 ± 3.70		29 ± 3.60		29.60 ± 3.70
								Weight	83.20 ± 11.10		81.7 ± 11.1		82.90 ± 13.90
Dasgupta et al. (19)	NM	43	32 ± 10.5	14	Unknown	Healthy subjects	↓	BMI	22.68 ± 4.965.0			21.65 ± 4.75	
								Weight	51.60 ± 12.03			49.26 ± 11.55	
								WC	83.63 ± 11.01			81.32 ± 10.64	
Devendra et al. (12)	United Kingdom	52	62.3 ± 9.8	65.4	13 h 58 min	T2D patients	NA	Weight	87.60 ± 8.11			88.20 ± 8.48	
Ebrahimi et al. (8)	Iran	42	38 ± 7	59.5	16 h 22 min	Non-alcoholic fatty liver disease patients	NA	BMI	30.09 ± 4.49			29.28 ± 4.14	
								Weight	83.65 ± 13.02			81.50 ± 12.8	
								FP	34.70 ± 8.76			34.02 ± 8.82	
								WC	100.21 ± 11.02			99.26 ± 10.86	
								HC	107.02 ± 7.11			106.47 ± 7.09	
Elfert et al. (43)	Egypt	216	NM	65.3	14 h 18 min	Liver Cirrhosis Patients	NA	BMI	28.7 ± 3			27.30 ± 3	
Faris et al. (9)	UAE	57	36 ± 12.5	61.4	15 h	Overweight/ obese subjects	↑	BMI	30.80 ± 5.10		30.40 ± 5.10		
								Weight	89.40 ± 14.90		88.20 ± 14.50		
								FM	27 ± 9.10		26.10 ± 9.20		
								WC	93 ± 13		92 ± 12		
								HC	106 ± 11		105 ± 11		
								TBW	43.7 ± 8		43.3 ± 8		
								MM	55 ± 9.70		54.50 ± 6.30		
Faris et al. (20)	Jordan	50	32 ± 9.5	42	14 h 30 min	Healthy subjects	↓	BMI	26.30 ± 5.01	25.85 ± 4.91			26.40 ± 5
								Weight	71.82 ± 13.41	70.58 ± 13.20			71.92 ± 13.50
								FP	24.12 ± 12.60	20.38 ± 11.32			30.48 ± 11.32
								WC	83.62 ± 11.17	82.69 ± 10.34			82.68 ± 10.37
								HC	102.61 ± 9.66	101.9 ± 9.98			101.02 ± 9.67
								WHR	0.81 ± 0.07	0.81 ± 0.07			0.81 ± 0.06
Feizollahzadeh et al. (44)	NM	70	48 ± NM	100	Unknown	Healthy subjects	NA	BMI	27.98 ± 1.38			27.35 ± 1.51	
								Weight	79.77 ± 8.95			77.93 ± 8.93	
Finch et al. (45)	United Kingdom	41	35.3 ± 11.5	37	11 h 36 min	Healthy subjects	NA	Weight	71 ± 12.04		70.7 ± 12.80		70.8 ± 12.61
Gholami et al. (13)	Iran	20	NM	44.4	15 h 33 min	T2D patients	NA	BMI	28.53 ± 3.90			28.14 ± 3.92	
								WC	99.66 ± 10.30			100.33 ± 9.70	
Hassanein et al. (46)	NM	1,749	58.3 ± 13.1	55.6	Unknown	T2D patients	NA	Weight	84.70 ± 16				84 ± 14.40
								WC	100.30 ± 14.30				99.20 ± 14.40
Hassanein et al. (47)	United Kingdom	59	NM	55.9	Unknown	T2D patients	NA	Weight	77.34 ± 12.85				77.58 ± 13.53
Khan et al. (48)	Pakistan	35	21 ± 4	51.4	15 h 21 min	Healthy subjects	NA	BMI	21.33 ± 3.99		21.27 ± 4.03		21.25 ± 3.94
								Weight	60.49 ± 14.74		60.46 ± 15.02		60.17 ± 14.52
								WC	79.9 ± 10.18		79.74 ± 10.33		79.42 ± 10.87
								HC	95.56 ± 9.73		96.12 ± 7.69		96.19 ± 8.57
								WHR	0.84 ± 0.07		0.83 ± 0.07		0.82 ± 0.07
Khattak et al. (49)	Malaysia	30	NM	100	13 h 44 min	Healthy subjects	NA	BMI	23.50 ± 3.96	23.40 ± 4.01			
								Weight	69 ± 8.30	68 ± 8.40			
								WHR	0.85 ± 0.04	0.84 ± 0.05			
Karatoprak et al. (14)	Turkey	76	57.4 ± 10.1	25	15 h 30 min	T2D patients	NA	Weight	82.60 ± 14.60			82.70 ± 14.1	
Kiyani et al. (50)	Pakistan	80	20.5 ± NM	37.5	14 h 25 min	Healthy subjects	NA	Weight	62.70 ± 8.80			62.30 ± 9	
Muhammad et al. (51)	Indonesia	45	33 ± 11.5	28.9	13 h 30 min	Overweight/ obese subjects	↓	BMI	28.50 ± 3.50		27.90 ± 3.5	28.30 ± 3.70	
								Weight	71.30 ± 10.50		69.90 ± 10.4	70.90 ± 10.80	
								FP	33.70 ± 5.50		32.60 ± 5.6	33.20 ± 5.80	
								WC	85.20 ± 8.90		85.30 ± 10.5	85.60 ± 10.1	
Madkour et al. (52)	UAE	56	36 ± 12.4	60.7	15 h	Overweight/obese subjects	—	BMI	30.91 ± 5.21		30.45 ± 5.09		
								Weight	89.39 ± 14.87		88.24 ± 14.56		
								FM	27.31 ± 9.56		26.09 ± 9.24		
								FP	30.47 ± 7.06		29.64 ± 7.11		
								MM	57.77 ± 10.22		57.37 ± 10.08		
								WC	95 ± 13.27		91.9 ± 11.37		
								HC	105.91 ± 10.54		104.65 ± 10.42		
								TBW	43.5 ± 7.6		43.23 ± 7.5		
								WHR	0.89 ± 0.08		0.89 ± 0.07		
Malekmakan et al. (53)	Iran	93	37 ± 7.9	52.7	15 h 15 min	Healthy subjects	NA	BMI	26.1 ± 3.30			25.7 ± 3.20	
								Weight	71.6 ± 12.40			70.4 ± 12	
								WC	89.1 ± 11.10			87.5 ± 11.10	
								HC	101.4 ± 8.6			100.1 ± 8.50	
Khaled and Belbraouet (54)	Algeria	276	49 ± 6	0	12 h 29 min	Diabetic and obese	NA	BMI	35.16 ± 3.63	34.4 ± 3.64			35.14 ± 3.94
								Weight	84.26 ± 8.84	81.84 ± 8.67			83.6 ± 9.23
								WC	107.3 ± 8.06	106.89 ± 7.96			107.25 ± 8.01
								WHR	0.90 ± 0.06	0.89 ± 0.05			0.90 ± 0.05
Nachvak et al. (16)	Iran	152	39 ± 10.7	100	15 h 33 min	Healthy subjects	↓	BMI	26.10 ± 3.79		25.37 ± 3.74		26.08 ± 3.81
								Weight	76.33 ± 11.40		74.22 ± 11.20		76.31 ± 11.50
								FM	18.34 ± 6.10		17.60 ± 6.20		19.36 ± 6.20
								FP	23.50 ± 5.60		23.13 ± 5.80		24.80 ± 5.40
								WHR	0.9 ± 0.081		0.89 ± 0.082		0.90 ± 0.078
Namaghi et al. (55)	Iran	80	45.4 ± 9	68	15 h 55 min	Individuals with metabolic syndrome	↓	BMI	30.70 ± 4			30.10 ± 4	
								Weight	85.50 ± 13			84 ± 13	
								FM	31 ± 10			29.80 ± 10	
								MM	30.50 ± 6			30.20 ± 6	
								TBW	40.2 ± 7			39.9 ± 7	
Nematy et al. (56)	Iran	82	54 ± 10	46.3	13 h 44 min	Cardiovascular Patients	NA	BMI	28.40 ± 4			27.70 ± 4	
								Weight	73.50 ± 13			71.70 ± 13	
								WC	98.3 ± 9			96.70 ± 9	
Norouzy et al. (57)	Iran	82	40.1 ± 6.3	65.8	13 h 44 min	Healthy subjects	↓	BMI	26.18 ± 3.88			25.90 ± 3.85	
								Weight	71.81 ± 13.08			70.72 ± 13.01	
								FM	20.70 ± 6.16			20.26 ± 6.27	
								FP	28.52 ± 5.68			28.29 ± 6.05	
								WC	92.04 ± 11.01			90.71 ± 10.79	
								HC	102.93 ± 6.67			100.89 ± 6.83	
Norouzy et al. (58)	Iran	88	51 ± 10	51.1	13 h 44 min	T2D patients	NA	BMI	27.60 ± 3.90			27.20 ± 3.80	
								Weight	72.60 ± 12.90			70.90 ± 12.50	
								WC	97.90 ± 9.60			97.20 ± 9.60	
								HC	105.50 ± 7.90			104.70 ± 8.50	
Ongsara et al. (17)	Thailand	65	20.8 ± 1.1	63.7	13 h 56 min	Healthy subjects	NA	BMI	21.49 ± 3.55		21.27 ± 3.48		21.45 ± 3.61
								Weight	55.73 ± 12.99		55.06 ± 13.27		55.65 ± 13.20
								FM	12.68 ± 6.40		12.81 ± 6.52		14.21 ± 7.64
								FP	23.86 ± 7.40		22.71 ± 6.45		24.65 ± 7.33
								WC	72.31 ± 10.44		68.94 ± 9.78		69.84 ± 10.10
								MM	39.74 ± 8.91		39.83 ± 8.61		39.20 ± 7.88
Patel et al. (59)	NM	334	54.3 ± 11.7	43.7	Unknown	T1D patients	NA	BMI	28.90 ± 5.90			28.70 ± 5.90	
								Weight	70.60 ± 16			70.10 ± 16	
Sahin et al. (60)	NM	88	57 ± 9.6	32.8	Unknown	T2D patients	NA	BMI	36.32 ± 15.33			35.71 ± 11.47	
								Weight	89.96 ± 17.20			89.22 ± 16.68	
								WC	106.97 ± 15.57			106.06 ± 14.04	
Shariatpanahi et al. (61)	Iran	55	34.1 ± 8.9	100	15 h 54 min	Individuals with metabolic syndrome	NA	BMI	27.62 ± 3.39			26.87 ± 3.36	
								Weight	80.69 ± 12.27			78.73 ± 12.05	
								WC	94.81 ± 7.80			91.98 ± 7.70	
Shehab et al. (62)	UAE	60	38.7 ± 10.5	68	14 h 19 min	Healthy subjects	NA	BMI	27.83 ± 4.85		27.56 ± 4.75		27.76 ± 4.81
								Weight	78.58 ± 16.10		77.63 ± 15.89		78.17 ± 16.14
								WC	91.71 ± 14.14		89.31 ± 14.55		90.35 ± 14.08
Sulu et al. (15)	Turkey	45	28.7 ± NM	51.1	16 h 22 min	Healthy subjects	NA	BMI	26.2 ± 5.20		26.1 ± 5.30		
Syam et al. (63)	Indonesia	43	34 ± 11.3	16	13 h 11 min	Healthy subjects	↓	BMI	23.71 ± 3.90		23.35 ± 3.96		
								Weight	59.82 ± 11.25		58.95 ± 11.20		
								FM	17 ± 6.42		16.52 ± 6.33		
								TBW	30.81 4.9		30.5 ± 4.8		
Ghania et al. (64)	NM	80	56 ± 8	38.8	Unknown	T2D patients	NA	BMI	28.52 ± 4.44	28.83 ± 4.48			
								Weight	77.11 ± 11.96	77.73 ± 11.58			
								WC	95.98 ± 10.42	96.39 ± 10.25			
Toony et al. (65)	Egypt	200	50 ± 9.8	27	15 h 10 min	T2D patients	NA	BMI	32.27 ± 6.30	32.82 ± 6.30			32.83 ± 6.30
								Weight	79.24 ± 14.50	78.67 ± 16.20			79.29 ± 14.5
								WC	99.17 ± 13.10	99.02 ± 13			98.82 ± 12.90
Yarahmadi et al. (66)	Iran	57	NM	29.8	11 h 18 min	T2D patients	NA	BMI	28.80 ± 4.56	27.35 ± 4.03	28.83 ± 4.99		
								WHR	0.867 ± 0.77	0.865 ± 0.79	0.866 ± 0.77		
Shariatpanahi et al. (67)	Iran	65	40.1 ± 10.8	100	Unknown	Individuals with metabolic syndrome	NA	BMI	28.62 ± 4.79			27.87 ± 3.34	
								WC	96.41 ± 8.22			93.80 ± 6.40	
Khan et al. (48)	Pakistan	75	52.8 ± 8.5	50.7	13 h 37 min	T2D patients	NA	Weight	71.43 ± 15.45		69.41 ± 11.55		70.16 ± 11.37
								WC	98.16 ± 10.13		97.84 ± 9.24		98.8 ± 8.82
								HC	102.26 ± 10.42		103.79 ± 10.65		103.69 ± 10.08
								WHR	0.96 ± 0.07		0.95 ± 0.07		0.96 ± 0.08
Imtiaz et al. (68)	Pakistan	34	47.7 ± 14.6	64.7	15 h	CKD patients	NA	Weight	59.30 ± 13.40		59.4 ± 13.70		
Laajam (69)	Saudi Arabia	39	51.5 ± 10.3	25.6	Unknown	T2D patients	NA	Weight	77.10 ± 18.17			76.50 ± 18.11	
Pathan and Patil (70)	India	30	NM	100	Unknown	Healthy subjects	NA	Weight	61.90 ± 11.39			60.56 ± 10.74	
Traore et al. (71)	Mali	25	48.5 ± 6.8	44	13 h 46 min	T2D patients	NA	Weight	84.20 ± 10.20		83 ± 9.80		83.10 ± 10.20
Abdullah et al. (72)	Yemen	98	NM	100	14 h 22 min	T2D patients	NA	BMI	24.2 ± 4.3		24.16 ± 4.18		
								WC	94.4 ± 11.3		94.3 ± 11.25		
Al-Rawi et al. (73)	UAE	57	38.4 ±	70.1	15 h 13 min	Overweight/obese subjects	—	Weight	88.3 ± 16.2			86.7 ±15.7	
								BMI	29.9 ± 5.02			29.4 ± 4.9	
								WC	98.6 ± 13.7			97.2 ± 13	
								HC	110.1 ± 9.5			108.6 ± 8.9	
								TBW	44 ± 7.3			43.8 ± 7	
								FP	29.5 ± 7.1			28.6 ± 7.3	
								FM	26.5 ± 9.5			25.3 ± 9.4	
								FFM	61.8 10.4			60.9 ± 11	
								MM	58.7 9.9			58.4 ± 9.6	
Das et al. (74)	India	75	NM	100	Unknown	Healthy subjects	NA	Weight	57.6 ± 15.13				62 ± 15.1
								BMI	24.1 ± 6.4				26 ± 6.3
Farag et al. (75)	Iraq	120	37.5 ± 6.6	50	15 h 19 min	hypertensive patients	NA	Weight	82.9 ± 15.6			79.4 ± 15.6	
								BMI	32.9 ± 5.1			31.5 ± 5.3	
								WC	108.8 ± 10.12			106.3 ± 10.1	
Gad et al. (76)	Egypt	40	46 ± 9	32.5	14 h 22 min	Non-alcoholic fatty liver disease patients	NA	BMI	30.9 ± 2.42			29.4 ± 1.93	
Harbuwono et al. (77)	Indonesia	37	53.05 ± 6.9	45.9	13 h 9 min	T2D patients	NA	Weight	65.41 ± 11.46				65.6 ± 11.9
								BMI	26.77 ± 4.52				26.76 ± 4.65
Ismail et al. (78)	Egypt	300	54.23 ± 8.8	44	15 h 10 min	T2D patients	NA	BMI	28.59 ± 3.69				27.9 ± 3.39
Jahrami et al. (79)	Bahrain	50	43.28 ± 10	100	14 h 40 min	Diagnosed with major depressive disorder	NA	Weight	82.04 ± 12.19			80.6 ± 12.25	
								BMI	29.47 ± 6.45			28.9 ± 6.39	
								WC	93.2 ± 10.2			92.62 ± 10.3	
								HC	100.76 ± 11.05			100.7 ± 11.1	
								WHR	0.93 ± 0.16			0.93 ± 0.17	
								FP	29.48 8.56			28.74 ± 8.42	
								FM	29.9 ± 9.31			30.92 ± 2.88	
López-Bueno et al. (80)	North Africa	62	33.6 ± 12.7	0	15 h 26 min	Healthy subjects	NA	Weight	67.2 ± 14.1		66.1 ± 14		
								BMI	26.3 ± 5.8		25.8 ± 5.6		
								WC	90.1 ±12.4		89.4 ±12.4		
								HC	99 ± 12.7		98.1 ± 12.7		
								TBW	31.3 ± 3.4		31.1 ± 3.3		
								FP	32.1 ± 9.4		31.4 ± 9.5		
								FM	22.8 ± 10.7		22 ± 10.5		
								WHR	0.9 ± 0		0.9 ± 0		
								MM	42.2 ± 3.9		42.3 ± 3.8		
Mohamed et al. (81)	Saudi Arabia	289	42.3 ± 12.4	60.6	14 h 12 min	T2D patients	NA	BMI	28.3 ± 6.27				27.43 ± 5.92
Mohammadzadeh et al. (82)	Iran	30	41.57 ± 2.5	80	10 h 51 min	Healthy subjects	NA	BMI	25.72 ± 3.17			25.25 ± 3.01	
Urooj et al. (83)	India	52	37.8 ± 12	48	12 h	Healthy subjects	NA	Weight	71.65 ± 14.43			70.96 ± 14	
								BMI	27.45 ± 4.52			27.21 ± 3.18	
								FP	36.11 ± 10.24			36.36 ± 9.9	
								WHR	0.914 ± 0.06			0.92 ± 0.05	
								MM	30.5 ± 3.7			30.1 ± 3.6	
Yazdanyar et al. (84)	Iran	40	55.23 ± 9.3	17.5	15 h 53 min	T2D patients	NA	Weight	74.9 ± 12.7			73 ± 13.1	
								BMI	29.9 ± 5.2			29 ± 5.1	
								WC	106.2 ± 11.8			105.3 ± 11.6	
								WHR	0.98 ± 0.05			0.97 ± 0.04	
LORANS 2019	UK	82	45.4 ± 16	52.9	17 h 10 min	General population	—	BMI	28.37 ± 4.84			27.61 ± 4.32	
								Weight	76.57 ± 13.60			74.58 ± 12.23	
								FM	23.39 ± 10.60			22.48 ± 10.05	
								FP	30.62 ± 11.03			30.09 ± 10.91	
								WC	95.88 ± 13.07			93.82 ± 11.87	
								HC	106.50 ± 9.57			102.32 ± 8.70	
								TBW	37.78 ± 7.86			35.8 ± 7.21	

NM, not mentioned; NA, not applicable; ^*^= whether caloric intake during Ramadan increased (**↑**), decreased (**↓**) or didn't change (**—**) compared to the month before or after.

#### Effect on BMI and weight

**Figure 3:**
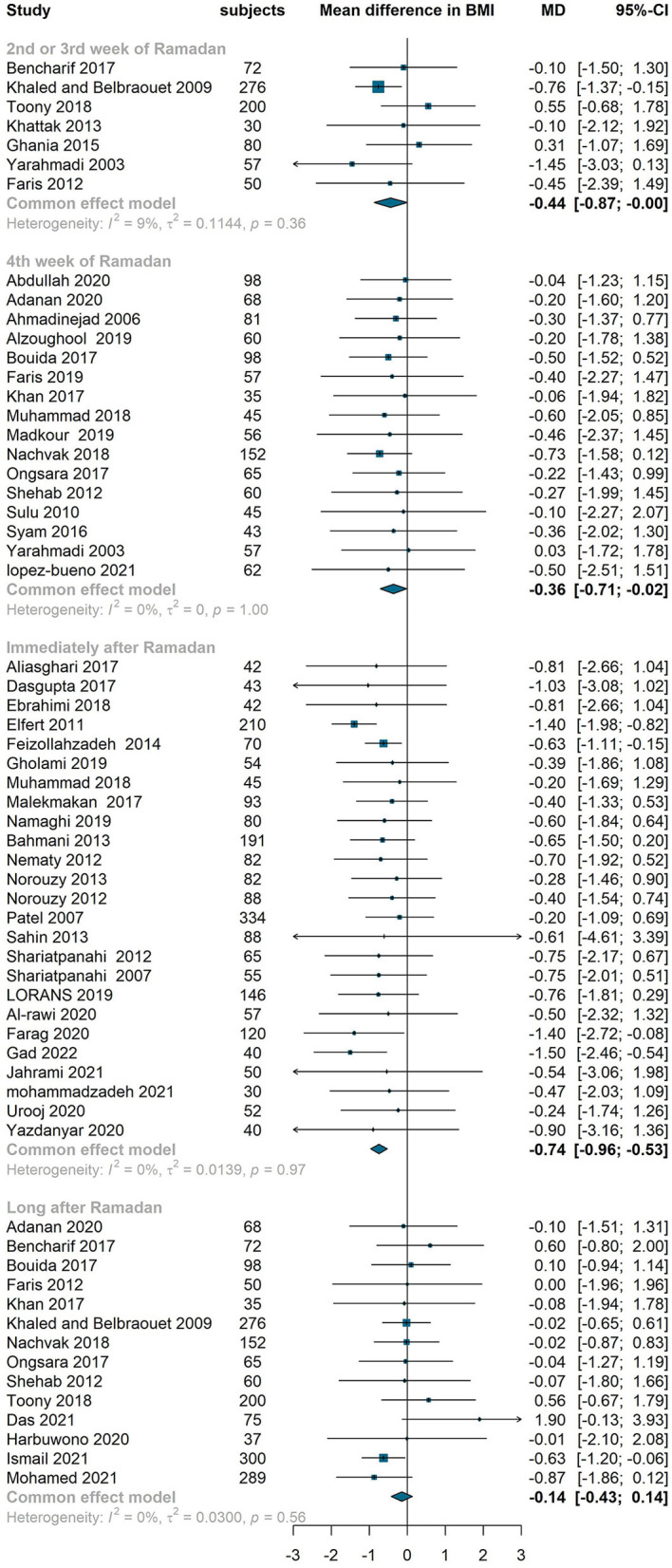
Fixed effect meta-analysis of RIF effect on BMI.

**Figure 4:**
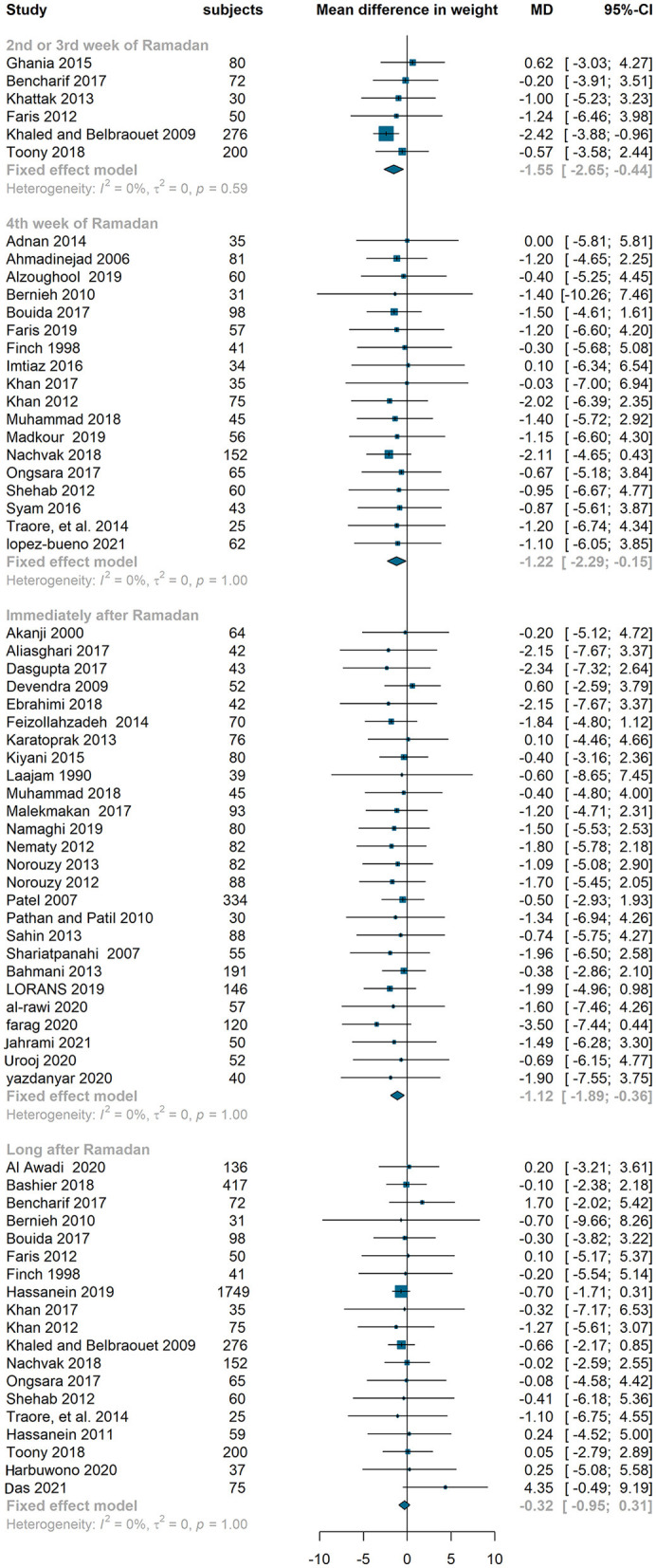
Fixed effect meta-analysis of RIF effect on weight.

We observed a similar pattern (a drop at the end of Ramada/immediately after Ramadan and a rise long after Ramadan) in BMI and weight. Compared to baseline values, BMI reduced at middle of Ramadan by 0.44 kg/m^2^ (95 % confidence interval −0.87 to 0, I^2^ = 9%), end of Ramadan by 0.36 kg/m^2^ (−0.71 to −0.02, I^2^=0%) and immediately after Ramadan by 0.74 kg/m^2^ (−0.96 to −0.53, I^2^=0%). However, the mean difference of BMI between baseline and long after Ramadan was not significant (−0.14 kg/m^2^, −0.43 to 0.14, I^2^=0%) (Figure 3). The similar pattern was observed in weight which decreased significantly at middle, end and immediately after Ramadan (−1.55 kg, −2.65– −0.44, I^2^ = 0%; −1.22 kg, −2.29– −0.15, I^2^ = 0%; −1.12 kg, −1.89– −0.36, I^2^ = 0%, respectively) but the reduction was not significant long after Ramadan (−0.32 kg, −1.95–0.31, I^2^ = 0%) (Figure 4).

#### Effect on fat, muscle and body water

At the end of Ramadan and immediately after Ramadan, there was a drop in both FP (−0.66 kg, −1.57–0.25, I^2^ = 0%; −0.46 kg, −1.45–0.53, I^2^ = 0%) and FM (−0.64 kg, −1.54–0.26, I^2^ = 0%; −0.49 kg, −1.62–0.65, I^2^ = 0%). Long after Ramadan, FP (1.15 kg, 0.14–2.16, I^2^ = 32%) and FM (0.95 kg, −0.15–2.06, I^2^ = 0%) were higher than the baseline values (Supplementary materials 9, 10). Muscle mass reduced by the end of Ramadan by −0.25 kg (−2.10–1.60, I^2^ = 0%) (Supplementary material 11). TBW was also reduced at the end of Ramadan by −0.25 kg (−1.16–0.67, I^2^ = 0%) and immediately after Ramadan by −1.09 kg (−2.29–0.11, I^2^ = 0%) (Supplementary material 12).

#### Effect on WC, HC and WHR

**Figure 5:**
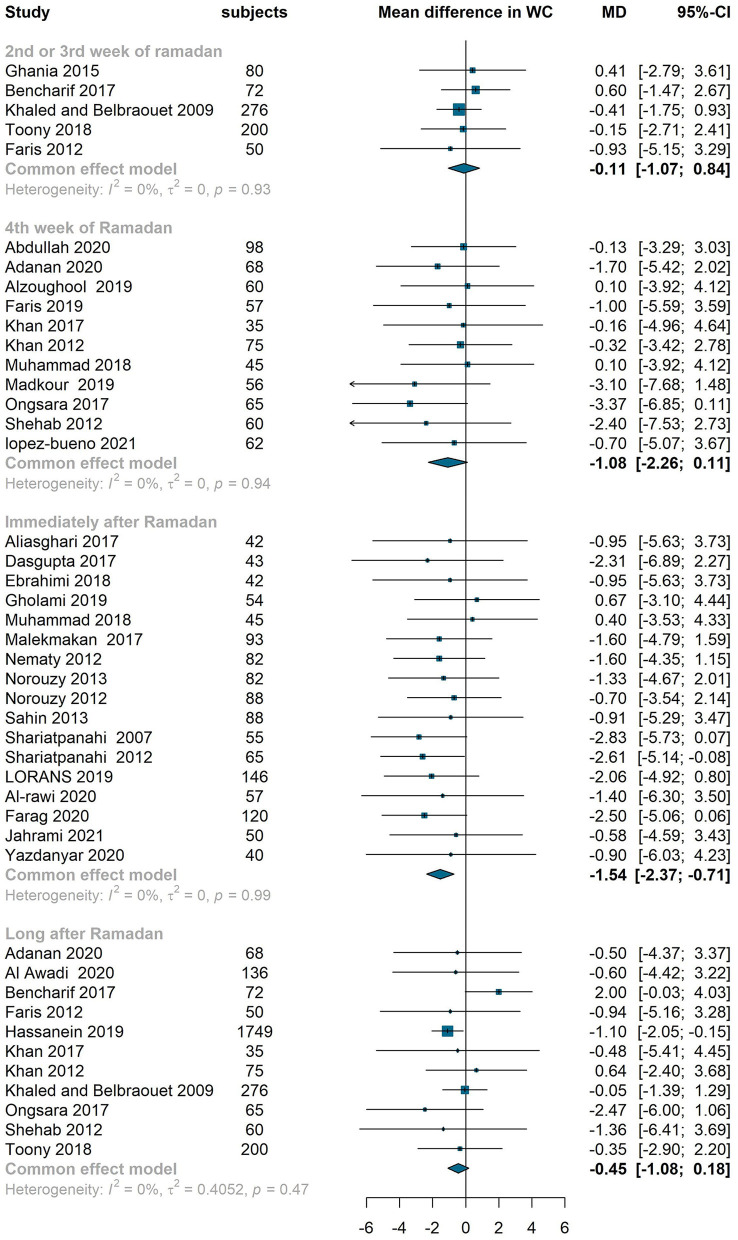
Fixed effect meta-analysis of RIF effect on WC.

WC, HC and WHR are also affected by RIF. The meta-analysis showed reductions compared to baseline in WC in the middle of Ramadan (−0.11 cm, −1.07–0.84, I^2^ = 0%), at the end of Ramadan (−1.08 cm, −2.26–0.11, I^2^ = 0%), immediately after Ramadan (−1.54 cm, −2.37– −0.71, I^2^ = 0%) but no significant difference long after Ramadan (−0.45 cm, −1.08–0.18, I^2^ = 0%) (Figure 5). HC did not change by the end of Ramadan (−0.07 cm, −1.82–1.69, I^2^ = 0%), decreased significantly immediately after Ramadan (−1.76 cm, −2.69– −0.83, I^2^ = 10%) and did not show a significant difference long after Ramadan (0.26 cm, −1.89–2.41, I^2^ = 0%) (Supplementary material 13). There was a negligible change in the waist to hip ratio at the middle, end and long after Ramadan (Supplementary material 14).

#### Small-study effects bias

To assess for asymmetry, we constructed funnel plots and ran Egger's test on all subgroups and considered a significant *p*-value as evidence for asymmetry (Supplementary material 15). The trim and fill method was applied to correct for the potential asymmetry. However, after applying this method, no changes were observed in the effects.

## Discussion

*LORANS* found that RIF is associated with a significant reduction in body weight, WC, HC, BMI, FP, FM, TBW, trunk FFM and trunk PMM immediately after Ramadan. Most of these changes were independent of physical activity, energy intake and extremities PMM but partially dependent on TBW and FM. The meta-analysis has 66 studies (including LORANS) and 7,496 participants indicated a significant reduction in weight, BMI, WC and HC starting within the second and third weeks of Ramadan, continuing in the fourth week of Ramadan, peaking immediately after Ramadan and declining to reach the pre-Ramadan levels 3 weeks or more after Ramadan.

Conducting *LORANS* enabled us to investigate the effect of adjustments on the changes in anthropometric and body composition indices of interest. The main adjustments that made a change in anthropometric changes after Ramadan were FM and TBW which decreased significantly after Ramadan and are more likely to be mediators rather than confounders. In other words, the observed changes in anthropometric and body composition measures are due to losing fat or dehydration. The latter should not be confused with daily dehydration which happens due to abstinence from water drinking during Ramadan since the measurements were done a few days after Ramadan when participants were supposed to be well-hydrated. Although some studies reported that RIF is associated with lower energy intake and physical activity (85, 86), the two factors didn't change significantly after Ramadan and didn't change the observed effects of RIF on anthropometric and body composition indices in *LORANS*. Fernando et al. (27) argued that decreased weight and fat might be due to increased energy expenditure, but another study showed that there is no association between RIF and energy expenditure (86).

Several animal and human studies on intermittent fasting reported reduced body fat and maintained muscle and attributed the observed effects to the metabolic switch (87–89). In this state, the body uses ketones and fatty acids for energy and the metabolism becomes more efficient (90). Although we reported similar effects, we are still uncertain about the mechanism behind the effects of RIF on anthropometric measurements and body composition due to the differences between RIF and the other types of intermittent fasting.

In 2022, Erdem et al. conducted a recent study (91) that assigned 360 individuals into five groups, including time-restricted fasting (TRF) with 6 h eating window (TRF-6 h), TRF with 8 h eating window (TRF-8 h) and alternate-day fasting (ADF). There was no restriction on calorie intake in the two TRF models, but it was restricted to <500 kcal 2 days per week in ADF. They measured anthropometric measurements on three-time points (after 1, 6 and 12 weeks). Similarly to Ramadan effects in our study, the authors reported that the three fasting models significantly reduced weight, BMI, and WC but not HC. Interestingly, although energy intake in the two TFR groups was almost identical, the reductions in all indices were greater in group TRF-6 h than in group TRF-8 h. The eating window in the two TRF models is close to that in LORANS (~7 h). However, the eating window was at night in LORANS, but in Erdem et al. study was during daylight hours. Also, unlike RIF, no-calorie drinks were allowed in Erdem et al. study. RIF has no calorie restriction like ADF. In addition, the duration of exposure in LORANS (4 weeks) is not the same as in Erdem et al. (1 week, 6 weeks and 8 weeks). These differences make the direct comparison between RIF and the other three models of intermittent fasting (TRF-6 h, TRF-8 h and ADF) imprecise. However, the anthropometric measurements' reductions in LORANS were half or less than that reduced in the other three models after 6 weeks. Furthermore, a systematic review and meta-analysis (24) compared the effect of RIF on anthropometric and body composition indices to that of non-RIF (all other types of intermittent fasting) combined. This review reported that both reduced the indices, but the non-RIF had a greater effect.

Although this review is the only one that investigated effects on the general population and didn't focus on a particular group of participants, most of our findings are consistent with other reviews. The significant reduction observed in weight during the fourth week and immediately after Ramadan is compatible with four of the previous systematic reviews (21–24, 27). Likewise, the decrease in WC was similar to the decrease reported by Faris et al. (26). For BMI, however, a meta-analysis by Aydin et al. (25) reported no significant decrease in BMI, whereas we observed a significant decrease immediately after Ramadan. This difference is due to the fact that the pooled estimate from Aydin et al. included a wide range of measurement times from the last week of Ramadan up to 6 weeks after Ramadan. We also reported that the differences were not significant long after Ramadan. It is possible that the overall effect estimates are diluted in Aydin et al. and did not reach the significance level. Another review (27) that reported a meta-analysis of data from studies on healthy and overweight/obese individuals showed a significant decrease in FM and FP during the fourth week and immediately after Ramadan followed by a return to baseline levels long after Ramadan. In that meta-analysis, the authors included more studies on overweight/obese individuals than studies on individuals with a healthy weight. This suggests that, compared to normal weight people, overweight/obese individuals lose more FM and FP during Ramadan. However, this was not the case in LORANS.

The bioelectrical impedance analyser depends on tissues electrical conductivity which varies based on their water content (92). It requires information for each participant (i.e., sex and age) to use an appropriate equation to predict body composition parameters (93). However, BIA uses constant values for density and hydration of FFM and assumes that they are similar in all individuals when estimating FFM (94), then it predicts FM based on the estimated FFM (92). Also, calculated parameters by BIA are not identical to some other reference methods that have higher precision such as DEXA (93). Although BIA may underestimate or overestimate FP (30), it's precision is high enough to be used in epidemiological studies (95). Using DEXA in *LORANS* would be impractical, and BIA appeared to be the best method to be used due to the study nature, being operator-independent and its high precision (30, 95–98).

This study has several strengths. *LORANS* is a community-based study while all previous studies didn't target the general population. Also, this is the only systematic review and meta-analysis conducted to observe the effect of RIF on anthropometric measurements and body composition in the general population rather than in specific groups such as healthy individuals (21–23, 26, 27) or T2D patients (25). For the first time, we conducted a meta-analysis on the effect of RIF at four different time points of follow-up which allowed us to make conclusions on the reversibility of the changes in anthropometry and body composition after Ramadan. Moreover, *LORANS* allowed us to investigate the anthropometric and body composition changes independent of changes in energy intake as well as physiological changes such as TBW and FM. A number of limitations should also be acknowledged. First, the second visit in LORANS was 8–12 days after Ramadan, meaning that the lifestyle changes during these days interfered with the effect of RIF. Another limitation in LORANS is the 41.7% drop-out in the baseline sample. Also, in the meta-analysis, we did not find many studies that have studied indices such as MM, so it was not possible to conduct separate meta-analyses at different time points. Moreover, not all indices were measured at all time points. Thus, the meta-analysis for various time points included different studies which reduced the comparability of the time points. Finally, we observed an indication of small-studies bias in six of the subgroups. However, applying the trim and fill method on these subgroups showed that the effect of bias is minimal.

In the general population, RIF is associated with reductions in weight, BMI, WC and HC. These reductions tend to start at the second/third week of Ramadan, become significant in the fourth week, reach their maximum immediately after Ramadan, and fade away 3–6 weeks after Ramadan. Reductions in TBW and FM might drive the changes in anthropometric and body composition changes during and after Ramadan.

## Author contributions

RA-J, AD, and KT conceptualized the paper and interpreted data analysis. RA-J and AD collected data. RA-J, NW, KB, ME, ZB, ZA, and AA screened abstracts and full-texts. RA-J, NW, ME, and ZB extracted data and assessed the quality of reviewed studies. AD, KT, and PE critically reviewed the manuscript and provided feedback. RA-J is the guarantor, wrote the manuscript, and attests that all listed authors meet authorship criteria and that no others meeting the criteria have been omitted. All authors contributed to the article and approved the submitted version.

## Supplementary material

Supplementary material 1 Characteristics of individuals who did not attend the second visit after Ramadan compared to LORANS participants.

Supplementary material 2 PRISMA checklist.

Supplementary material 3 Newcastle-Ottawa quality assessment scale (Adapted).

Supplementary material 4 Quality assessment of potential.

Supplementary material 5 Characteristics of LORANS' participants (*n* = 146).

Supplementary material 6 Effect of adjustments on mean difference in anthropometric and body composition parameters from LORANS.

Supplementary material 7 Effect of RIF on anthropometric and body composition parameters on LORANS participants grouped by baseline BMI classes.

Supplementary material 8 The included studies in the meta-analysis with measured parameters and time-point of measurement.

Supplementary material 9 Fixed effect meta-analysis of RIF effect on fat percentage.

Supplementary material 10 Fixed effect meta-analysis of RIF effect on fat mass.

Supplementary material 11 Fixed effect meta-analysis of RIF effect on muscle mass during the fourth week of Ramadan.

Supplementary material 12 Fixed effect meta-analysis of RIF effect on total body water.

Supplementary material 13 Fixed effect meta-analysis of RIF effect on hip circumference.

Supplementary material 14 Fixed effect meta-analysis of RIF effect on the waist to hip ratio.

Supplementary material 15 Risk of bias tests.
