# Supplementary material for: The impact of Ramadan intermittent fasting on anthropometric measurements and body composition: Evidence from LORANS study and a meta-analysis
Source: Front Nutr. 2023 Jan 17;10:1082217. doi: 10.3389/fnut.2023.1082217 (PMC9886683; doi:10.3389/fnut.2023.1082217)
Supplement: Supplementary material 1 — Characteristics of individuals who did not attend the second visit after Ramadan compared to LORANS participants. [file Data_Sheet_1.zip › SM1.docx]

**Supplementary Material 1**: characteristics of individuals who did not attend the second visit after Ramadan compared to LORANS participants.

| Variable | Sub-groups | Study participants (n=85) | Individuals who didn’t attend the second visit (n=61) | p-value |
| --- | --- | --- | --- | --- |
| Age  (mean ± SD) | Total | 45.6 ± 15.9 | 39 ± 21.3 ^꙰^ | 0.002 |
|  | 18 – 40 years (%) | 31.8% | 55.7% |  |
|  | 40 – 60 years (%) | 49.4% | 39.3% |  |
|  | 60 – 80 years (%) | 17.6% | 11.5% |  |
|  | > 80 years (%) | 1.2% | 3.3% |  |
| Sex  (male %) | | 52.9% | 49.2 % | 0.80 |
| Ethnic background  (%) | Pakistani | 16.5% | 18.8% | 0.36 |
|  | Indian | 26.6% | 35.4% |  |
|  | Bangladeshi | 8.9% | 6.3% |  |
|  | Somali | 16.5% | 22.4% |  |
|  | Arab | 16.5% | 4.2% |  |
|  | Other | 15.2% | 12.5% |  |
| Marital status  (%) | Single | 22.8% | 32% | 0.49 |
|  | Married/living with a partner | 73.4 % | 66% |  |
|  | Divorced/separated | 3.8 % | 2.1% |  |
| With Chronic diseases  (%) | Diabetes | 25.9% | 13.1% | 0.83 |
|  | Hypertension | 44.7% | 13.1% | 0.48 |
|  | Cardiovascular diseases | 9.4% | 6.6% | 0.42 |
| Education  (%) | No formal qualification | 12.7% | 10.6% | 0.31 |
|  | Secondary school or equivalent | 25.3% | 19.1% |  |
|  | Higher education: College/HNC/HND | 21.5% | 14.9% |  |
|  | Vocational qualification | 1.3% | 8.5% |  |
|  | Bachelor’s degree | 26.6% | 27.7% |  |
|  | Postgraduate degree | 12.7% | 19.1% |  |
| Smoking  (%) | Never | 77.6% | 91.5% | 0.02 |
|  | Stopped | 14.1% | 2.1% |  |
|  | Occasionally | 3.5% | 6.4% |  |
|  | Yes, most or all days | 4.7% | 0% |  |

^꙰ median^
