# Supplementary material for: The impact of Ramadan intermittent fasting on anthropometric measurements and body composition: Evidence from LORANS study and a meta-analysis
Source: Front Nutr. 2023 Jan 17;10:1082217. doi: 10.3389/fnut.2023.1082217 (PMC9886683; doi:10.3389/fnut.2023.1082217)
Supplement: Supplementary material 1 — Characteristics of individuals who did not attend the second visit after Ramadan compared to LORANS participants. [file Data_Sheet_1.zip › SM15.docx]

**Supplementary Material 15:** Risk of bias tests.

| **parameter** | **Time point** | **Egger test**  **p-value** | **p-value**  **using Trim & fill** | **MD**  **using trim & fill** | **Original**  **p-value** | **Original**  **MD** |
| --- | --- | --- | --- | --- | --- | --- |
| **BMI** | **2nd & 3rd week of R** | 0.33 | NA | NA | NA | NA |
|  | **Fourth week of R** | 0.05 | 0.002 | -0.47 | 0.04 | -0.36 |
|  | **immediately after R** | 0.25 | NA | NA | NA | NA |
|  | **Long after R** | 0.14 | NA | NA | NA | NA |
| **Weight** | **2nd & 3rd week of R** | 0.05 | < 0.001 | -2.2 | 0.05 | -1.55 |
|  | **Fourth week of R** | < 0.001 | 0.001 | -1.57 | 0.02 | -1.22 |
|  | **immediately after R** | 0.15 | NA | NA | NA | NA |
|  | **Long after R** | 0.06 | 0.06 | -0.6 | 0.21 | -0.32 |
| **WC** | **2nd & 3rd week of R** | 0.74 | NA | NA | NA | NA |
|  | **Fourth week of R** | 0.88 | NA | NA | NA | NA |
|  | **immediately after R** | 0.03 | < 0.001 | -2.02 | < 0.001 | -1.54 |
|  | **Long after R** | 0.75 | NA | NA | NA | NA |
| **HC** | **Fourth week of R** | 0.19 | NA | NA | NA | NA |
|  | **immediately after R** | 0.10 | NA | NA | NA | NA |
|  | **Long after R** | 0.75 | NA | NA | NA | NA |
| **FM** | **Fourth week of R** | 0.67 | NA | NA | NA | NA |
|  | **immediately after R** | 0.87 | NA | NA | NA | NA |
|  | **Long after R** | 0.76 | NA | NA | NA | NA |
| **FP** | **Fourth week of R** | 0.33 | NA | NA | NA | NA |
|  | **immediately after R** | 0.72 | 0.53 | -0.33 | 0.50 | −0.40 |
|  | **Long after R** | 0.69 | NA | NA | NA | NA |
| **WHR** | **2nd & 3rd week of R** | 0.49 | NA | NA | NA | NA |
|  | **Fourth week of R** | 0.69 | NA | NA | NA | NA |
|  | **immediately after R** | 0.94 | NA | NA | NA | NA |
|  | **Long after R** | 0.32 | NA | NA | NA | NA |
| **MM** | **Fourth week of R** | 0.81 | NA | NA | NA | NA |
| **TBW** | **Fourth week of R** | 0.60 | NA | NA | NA | NA |
