# Supplementary material for: The impact of Ramadan intermittent fasting on anthropometric measurements and body composition: Evidence from LORANS study and a meta-analysis
Source: Front Nutr. 2023 Jan 17;10:1082217. doi: 10.3389/fnut.2023.1082217 (PMC9886683; doi:10.3389/fnut.2023.1082217)
Supplement: Supplementary material 1 — Characteristics of individuals who did not attend the second visit after Ramadan compared to LORANS participants. [file Data_Sheet_1.zip › SM3.docx]

**Supplementary Material 3:** Newcastle-Ottawa quality assessment scale (Adapted)

**Selection**

**1) Representativeness of the sample:** (Maximum is 1 star)

a) Truly representative of the average in the target population. *

b) Somewhat representative of the average in the target population. *

c) Selected group of users.

d) No description of the sampling strategy.

**2) Sample size:**(Maximum is 1 star)

a) Satisfactory (≥100 OR ≥50 participants with power calculation). *

b) Unsatisfactory (<100 participants without power calculation OR < 50 participants).

**3) Non-respondents:**(Maximum is 1 star)

a) Comparability between respondents and non-respondents characteristics is established, and the response rate is satisfactory (response rate is ≥ 50%). *

b) The response rate is unsatisfactory, or the comparability between respondents and non-respondents is unsatisfactory (response rate is < 50%).

c) No description of the response rate or the characteristics of the responders and the non-responders.

**4) Ascertainment of the exposure (Ramadan fasting):** (Maximum is 2 stars)

a) Considered the number of days fasted by each participant. **

b) no details of the number of days fasted.*

**Comparability**

**1) The subjects in different outcome groups are comparable, based on the study design or analysis. Confounding factors are controlled:** (Maximum is 2 stars)

a) before/after design with control for main demographic factors by design. *

b) The study controls for any additional factor (physical activity, smoking or diet). *

**Outcome**

1. **Assessment of the outcome (Anthropometric  Measurement):** (Maximum is 1 star)

 a) no description

b) self reported

c) measured*

**2) Statistical test:** (Maximum is 1 star)

a) The statistical test used to analyze the data is clearly described and appropriate, and the measurement of the association is presented.*

b) The statistical test is not described or not appropriate.

We adapted this scale from the Newcastle-Ottawa Quality Assessment Scale for cohort studies (1)  to assess the quality of selected studies in this systematic review “**The effect of religious fasting in Ramadan on Anthropometric Measurement and body composition: Evidence from LORANS and a meta-analysis**” as has been done in a previous study (2).

1.Wells G, Shea B, O’Connell D, Peterson J, Welch V, Losos M, et al. The Newcastle-Ottawa Scale (NOS) for assessing the quality of nonrandomised studies in meta-analyses: The Ottawa Hospital Research Institute; 2019 [Available from: <http://www.ohri.ca/programs/clinical_epidemiology/oxford.asp>.

2.Herzog R, Alvarez-Pasquin MJ, Diaz C, Del Barrio JL, Estrada JM, Gil A. Are healthcare workers’ intentions to vaccinate related to their knowledge, beliefs and attitudes? A systematic review. BMC public health. 2013;13:154.
