# Supplementary material for: The impact of Ramadan intermittent fasting on anthropometric measurements and body composition: Evidence from LORANS study and a meta-analysis
Source: Front Nutr. 2023 Jan 17;10:1082217. doi: 10.3389/fnut.2023.1082217 (PMC9886683; doi:10.3389/fnut.2023.1082217)
Supplement: Supplementary material 1 — Characteristics of individuals who did not attend the second visit after Ramadan compared to LORANS participants. [file Data_Sheet_1.zip › SM4.docx]

**Supplementary Material 4:** quality assessment of potential.

| **Authors** | **Selection** | | | | **comparability** | **Outcome** | | **Score** | **quality** |
| --- | --- | --- | --- | --- | --- | --- | --- | --- | --- |
|  | Representativeness | Sample size | Non-respondents | exposure |  | Assessment of outcome | Statistical test |  |  |
| Adanan et al 2020 | 1 | 0 | 0 | 2 | 1 | 1 | 1 | 6 | Satisfactory |
| Adlouni et al 1997 | 0 | 0 | 0 | 1 | 1 | 1 | 1 | 4 | Low |
| Adlouni et al 1998 | 0 | 0 | 0 | 1 | 1 | 1 | 1 | 4 | Low |
| Adnan et al 2014 | 1 | 0 | 0 | 2 | 1 | 0 | 1 | 5 | Satisfactory |
| Ahmadinejad et al 2006 | 1 | 0 | 0 | 2 | 1 | 0 | 1 | 5 | Satisfactory |
| Akanji et al 2000 | 1 | 0 | 0 | 1 | 1 | 1 | 1 | 5 | Satisfactory |
| Akin et al 2020 | 1 | 0 | 0 | 1 | 1 | 1 | 0 | 4 | Low |
| Al Awadi et al 2020 | 1 | 1 | 0 | 2 | 1 | 0 | 1 | 6 | Satisfactory |
| Al-Barha et al 2018 | 0 | 0 | 0 | 1 | 1 | 1 | 1 | 4 | Low |
| Aliasghari et al 2017 | 1 | 0 | 0 | 1 | 1 | 1 | 1 | 5 | Satisfactory |
| Al-Numair et al 2006 | 0 | 0 | 0 | 1 | 1 | 1 | 1 | 4 | Low |
| Alzoughool et al 2019 | 1 | 0 | 0 | 1 | 1 | 1 | 1 | 5 | Satisfactory |
| Aybak et al 1996 | 0 | 0 | 0 | 1 | 1 | 0 | 1 | 3 | Low |
| Bahammam et al 2004 | 0 | 0 | 0 | 1 | 1 | 1 | 1 | 4 | Low |
| Bakiner et al 2009 | 0 | 0 | 0 | 1 | 1 | 1 | 1 | 4 | Low |
| Bashier et al 2018 | 1 | 1 | 0 | 2 | 1 | 0 | 1 | 6 | Satisfactory |
| Bernieh et al 2010 | 1 | 0 | 0 | 2 | 1 | 0 | 1 | 5 | Satisfactory |
| Bencharif 2017 | 1 | 0 | 0 | 2 | 1 | 1 | 1 | 6 | Satisfactory |
| Bilto et al 1998 | 0 | 0 | 0 | 1 | 1 | 1 | 1 | 4 | Low |
| Boobes et al 2009 | 0 | 0 | 0 | 1 | 1 | 0 | 1 | 3 | Low |
| Bouida et al 2017 | 1 | 0 | 0 | 1 | 1 | 1 | 1 | 5 | Satisfactory |
| Bueno et al 2015 | 0 | 0 | 0 | 1 | 1 | 1 | 1 | 4 | Low |
| Celik et al 2013 | 0 | 0 | 0 | 1 | 1 | 1 | 1 | 4 | Low |
| Ch'ng et al 1989 | 0 | 0 | 0 | 1 | 1 | 1 | 1 | 4 | Low |
| Culha et al 1970 | 0 | 0 | 0 | 1 | 1 | 1 | 1 | 4 | Low |
| Dasgupta et al 2017 | 1 | 0 | 0 | 1 | 1 | 1 | 1 | 5 | Satisfactory |
| Develioglu et al 2013 | 0 | 0 | 0 | 1 | 1 | 1 | 1 | 4 | Low |
| Devendra et al 2009 | 1 | 0 | 0 | 2 | 1 | 0 | 1 | 5 | Satisfactory |
| Dewanti et al 2006 | 1 | 0 | 0 | 1 | 1 | 0 | 1 | 4 | Low |
| Dwivedo et al 1996 | 0 | 0 | 0 | 1 | 1 | 1 | 1 | 4 | Low |
| Ebrahimi et al 2018 | 1 | 0 | 0 | 2 | 1 | 1 | 1 | 6 | Satisfactory |
| Elamin et al 2015 | 0 | 0 | 0 | 1 | 1 | 1 | 1 | 4 | Low |
| El-Ati et al 1995 | 0 | 0 | 0 | 1 | 1 | 1 | 1 | 4 | Low |
| Elfert et al 2011 | 1 | 1 | 0 | 1 | 1 | 0 | 1 | 5 | Satisfactory |
| Faris et al 2012 | 1 | 0 | 0 | 2 | 1 | 1 | 1 | 6 | Satisfactory |
| Faris et al 2019 | 0 | 1 | 0 | 2 | 1 | 1 | 1 | 6 | Satisfactory |
| Fedail et al 2014 | 0 | 0 | 0 | 1 | 1 | 0 | 1 | 3 | Low |
| Feizollahzadeh et al 2014 | 0 | 0 | 0 | 2 | 1 | 1 | 1 | 5 | Satisfactory |
| Finch et al 1998 | 1 | 0 | 0 | 1 | 1 | 1 | 1 | 5 | Satisfactory |
| Furuncuglo et al 2007 | 0 | 0 | 0 | 2 | 1 | 0 | 1 | 4 | Low |
| Gholami et al 2019 | 0 | 0 | 0 | 2 | 1 | 1 | 1 | 5 | Satisfactory |
| Haouari et al 2008 | 0 | 0 | 0 | 1 | 1 | 1 | 1 | 4 | Low |
| Hassanein et al 2019 | 1 | 1 | 0 | 2 | 1 | 1 | 1 | 7 | Good |
| Hassanein et al 2011 | 0 | 1 | 0 | 2 | 1 | 0 | 1 | 5 | Satisfactory |
| Hosseini et al 2013 | 0 | 0 | 0 | 1 | 1 | 1 | 1 | 4 | Low |
| Hourani et al 2007 | 0 | 0 | 0 | 1 | 1 | 1 | 1 | 4 | Low |
| Husain et al 1987 | 0 | 0 | 0 | 1 | 1 | 1 | 1 | 4 | Low |
| Ibrahim et al 2010 | 0 | 0 | 0 | 1 | 1 | 1 | 0 | 3 | Low |
| Imtiaz et al 2016 | 1 | 0 | 0 | 2 | 1 | 0 | 1 | 5 | Satisfactory |
| Iqbal et al 2019 | 0 | 0 | 0 | 1 | 1 | 1 | 1 | 4 | Low |
| J. Ramadan et al 2002 | 0 | 0 | 0 | 1 | 1 | 1 | 1 | 4 | Low |
| Justin et al 2015 | 0 | 0 | 0 | 1 | 1 | 1 | 1 | 4 | Low |
| Karatoprak et al 2013 | 1 | 1 | 0 | 1 | 1 | 1 | 1 | 6 | Satisfactory |
| Kassab et al 2003 | 0 | 0 | 0 | 1 | 1 | 1 | 1 | 4 | Low |
| Kassab et al 2004 | 0 | 0 | 0 | 1 | 1 | 1 | 1 | 4 | Low |
| Kayikcioglu et al 1999 | 0 | 0 | 0 | 1 | 1 | 1 | 1 | 4 | Low |
| Khaled et al 2006 | 0 | 0 | 0 | 1 | 1 | 0 | 1 | 3 | Low |
| Khaled et al 2009 | 0 | 0 | 0 | 1 | 1 | 1 | 1 | 4 | Low |
| Khan et al 2012 | 1 | 0 | 0 | 2 | 1 | 0 | 1 | 5 | Satisfactory |
| Khan et al 2017 | 1 | 0 | 0 | 2 | 2 | 1 | 1 | 7 | Good |
| Khattak et al 2013 | 1 | 0 | 0 | 1 | 1 | 1 | 1 | 5 | Satisfactory |
| Kiyani et al 2015 | 1 | 0 | 0 | 1 | 1 | 1 | 1 | 5 | Satisfactory |
| Laajam et al 1990 | 1 | 0 | 0 | 2 | 1 | 0 | 1 | 5 | Satisfactory |
| Latiri et al 2017 | 0 | 0 | 0 | 1 | 1 | 1 | 0 | 3 | Low |
| Muhammad et al 2018 | 1 | 0 | 0 | 1 | 1 | 1 | 1 | 5 | Satisfactory |
| M. Aakkoca et al 2018 | 0 | 0 | 0 | 1 | 1 | 1 | 1 | 4 | Low |
| Madkour et al 2019 | 0 | 1 | 0 | 1 | 1 | 1 | 1 | 5 | Satisfactory |
| Mafauzy et al 1990 | 1 | 0 | 0 | 1 | 1 | 0 | 1 | 4 | Low |
| Maislos et al 1998 | 0 | 0 | 0 | 1 | 1 | 1 | 1 | 4 | Low |
| Maislos et al 2001 | 0 | 0 | 0 | 1 | 1 | 1 | 1 | 4 | Low |
| Malekmakan et al 2017 | 1 | 1 | 0 | 2 | 1 | 1 | 1 | 7 | Good |
| Mansi & Amneh 2007 | 0 | 0 | 0 | 1 | 1 | 1 | 1 | 4 | Low |
| Mansi 2007 | 1 | 0 | 0 | 1 | 1 | 0 | 0 | 3 | Low |
| Mansoor et al 2019 | 0 | 0 | 0 | 1 | 1 | 1 | 1 | 4 | Low |
| Khaled and Belbraouet 2009 | 0 | 1 | 0 | 1 | 1 | 1 | 1 | 5 | Satisfactory |
| Mohajeri et al 2013 | 0 | 0 | 0 | 1 | 1 | 0 | 1 | 3 | Low |
| Nachvak et al 2018 | 0 | 1 | 0 | 1 | 1 | 1 | 1 | 5 | Satisfactory |
| Namaghi et al 2019 | 0 | 0 | 0 | 2 | 1 | 1 | 1 | 5 | Satisfactory |
| Nematy et al 2012 | 1 | 0 | 0 | 2 | 1 | 1 | 1 | 6 | Satisfactory |
| Norouzy et al 2012 | 1 | 1 | 0 | 2 | 1 | 1 | 1 | 7 | Good |
| Norouzy et al 2013 | 1 | 1 | 1 | 2 | 1 | 1 | 1 | 8 | Good |
| Nugraha et al 2017 | 0 | 0 | 0 | 1 | 1 | 1 | 1 | 4 | Low |
| Ongsara et al 2017 | 1 | 1 | 0 | 1 | 1 | 1 | 1 | 6 | Satisfactory |
| Pallayova et al 2017 | 0 | 0 | 0 | 1 | 1 | 1 | 1 | 4 | Low |
| Patel et al 2007 | 1 | 1 | 0 | 2 | 1 | 1 | 1 | 7 | Good |
| Pathan & Patil 2010 | 0 | 0 | 0 | 2 | 1 | 1 | 1 | 5 | Satisfactory |
| Prasetya et al 2018 | 0 | 0 | 0 | 1 | 1 | 1 | 1 | 4 | Low |
| Qadri et al 2005 | 1 | 0 | 0 | 1 | 1 | 1 | 0 | 4 | Low |
| Rahbar et al 2020 | 0 | 0 | 0 | 1 | 1 | 1 | 1 | 4 | Low |
| Rahman et al 2004 | 0 | 0 | 0 | 1 | 1 | 0 | 1 | 3 | Low |
| Rohin et al 2013 | 1 | 0 | 0 | 1 | 1 | 1 | 0 | 4 | Low |
| Alsubheen et al 2020 | 0 | 0 | 0 | 1 | 1 | 1 | 1 | 4 | Low |
| Saada et al 2009 | 0 | 0 | 0 | 1 | 1 | 1 | 1 | 4 | Low |
| Sahin et al 2013 | 1 | 1 | 0 | 2 | 1 | 0 | 1 | 6 | Satisfactory |
| Savas et al 2012 | 0 | 0 | 0 | 2 | 1 | 0 | 1 | 4 | Low |
| Sayedda et al 2013 | 0 | 0 | 0 | 1 | 1 | 1 | 1 | 4 | Low |
| Senhadji et al 2009 | 1 | 0 | 0 | 1 | 1 | 0 | 1 | 4 | Low |
| Sezen et al 2016 | 0 | 0 | 0 | 1 | 1 | 1 | 1 | 4 | Low |
| Shariatpanahi et al 2008 | 0 | 0 | 0 | 2 | 1 | 1 | 1 | 5 | Satisfactory |
| Shariatpanahi et al 2012 | 1 | 0 | 0 | 1 | 1 | 1 | 1 | 5 | Satisfactory |
| Shehab et al 2012 | 1 | 0 | 0 | 1 | 1 | 1 | 1 | 5 | Satisfactory |
| Subhan et al 2006 | 0 | 0 | 0 | 1 | 1 | 1 | 1 | 4 | Low |
| Sulimani et al 1999 | 1 | 0 | 0 | 1 | 1 | 0 | 1 | 4 | Low |
| Sulu et al 2010 | 1 | 0 | 0 | 1 | 1 | 1 | 1 | 5 | Satisfactory |
| Syam et al 2016 | 1 | 0 | 0 | 2 | 1 | 1 | 1 | 6 | Satisfactory |
| Talib et al 2015 | 0 | 0 | 0 | 1 | 1 | 1 | 1 | 4 | Low |
| Tashkandi et al 2019 | 1 | 0 | 0 | 1 | 1 | 0 | 1 | 4 | Low |
| Ghania et al 2015 | 1 | 0 | 0 | 2 | 1 | 1 | 1 | 6 | Satisfactory |
| Toony et al 2018 | 1 | 1 | 0 | 2 | 1 | 1 | 1 | 7 | Good |
| Traore et al 2014 | 1 | 0 | 0 | 1 | 1 | 1 | 1 | 5 | Satisfactory |
| Unalack et al 2011 | 0 | 0 | 0 | 1 | 1 | 1 | 1 | 4 | Low |
| Wakeel et al 2014 | 1 | 0 | 0 | 1 | 1 | 0 | 1 | 4 | Low |
| Yucel et al 2004 | 0 | 0 | 0 | 1 | 1 | 1 | 1 | 4 | Low |
| Zekri et al 2016 | 0 | 0 | 0 | 1 | 1 | 1 | 1 | 4 | Low |
| Bouguerra et al 2006 | 0 | 0 | 0 | 1 | 1 | 1 | 1 | 4 | Low |
| Martin et al 2004 | 0 | 0 | 0 | 1 | 1 | 1 | 1 | 4 | Low |
| Yarahmadi et al 2003 | 1 | 0 | 0 | 1 | 1 | 1 | 1 | 5 | Satisfactory |
| Bouguera et al 2003 | 0 | 0 | 0 | 1 | 1 | 0 | 1 | 3 | Low |
| Bahmani 2013 | 1 | 1 | 0 | 1 | 1 | 1 | 1 | 6 | Satisfactory |
| Zebidi et al 1990 | 0 | 0 | 0 | 0 | 1 | 1 | 1 | 3 | Low |
| Beltaifa et al 2002 | 0 | 0 | 0 | 2 | 1 | 0 | 1 | 4 | Low |
| Abdullah et al 2020 | 1 | 1 | 0 | 1 | 1 | 1 | 1 | 6 | Satisfactory |
| Al-Rawi et al 2020 | 0 | 1 | 1 | 2 | 1 | 1 | 1 | 7 | Good |
| Daldal et al 2021 | 0 | 0 | 0 | 2 | 1 | 0 | 1 | 4 | Low |
| Das et al 2021 | 0 | 1 | 1 | 1 | 1 | 1 | 1 | 6 | Satisfactory |
| Farag et al 2020 | 1 | 1 | 1 | 2 | 1 | 1 | 1 | 8 | Good |
| Gad et al 2022 | 0 | 0 | 0 | 2 | 1 | 1 | 1 | 5 | Satisfactory |
| Harbuwono et al 2021 | 1 | 0 | 0 | 2 | 1 | 1 | 1 | 6 | Satisfactory |
| Ismail et al 2021 | 1 | 1 | 0 | 1 | 1 | 1 | 0 | 5 | Satisfactory |
| Jahrami et al 2021 | 1 | 1 | 0 | 1 | 1 | 1 | 1 | 6 | Satisfactory |
| López-Bueno et al 2021 | 1 | 0 | 1 | 1 | 1 | 1 | 1 | 6 | Satisfactory |
| Mari et al 2020 | 0 | 0 | 1 | 1 | 1 | 0 | 1 | 4 | Low |
| Mohamed et al 2021 | 0 | 1 | 0 | 1 | 1 | 1 | 1 | 5 | Satisfactory |
| Mohammadzadeh et al 2021 | 1 | 0 | 0 | 2 | 1 | 1 | 1 | 6 | Satisfactory |
| Nassar et al 2020 | 0 | 0 | 0 | 2 | 1 | 1 | 0 | 4 | Low |
| Urooj et al 2020 | 1 | 0 | 1 | 2 | 1 | 1 | 1 | 7 | Good |
| Yazdanyar et al 2020 | 0 | 0 | 0 | 2 | 1 | 1 | 1 | 5 | Satisfactory |
| Bener et al 2021 | 0 | 1 | 1 | 1 | 1 | 0 | 0 | 4 | Low |
