# Supplementary material for: The impact of Ramadan intermittent fasting on anthropometric measurements and body composition: Evidence from LORANS study and a meta-analysis
Source: Front Nutr. 2023 Jan 17;10:1082217. doi: 10.3389/fnut.2023.1082217 (PMC9886683; doi:10.3389/fnut.2023.1082217)
Supplement: Supplementary material 1 — Characteristics of individuals who did not attend the second visit after Ramadan compared to LORANS participants. [file Data_Sheet_1.zip › SM5.docx]

**Supplementary Material 5**: Characteristics of LORANS’ participants (n=146).

| Variable | Sub-groups | Value |
| --- | --- | --- |
| Age  (mean ± SD) | Total | 43.3 ± 15 |
|  | 18 – 40 years (%) | 40.4% |
|  | 40 – 60 years (%) | 45.2% |
|  | 60 – 80 years (%) | 13.7% |
|  | > 80 years (%) | 0.7% |
| Sex  (male %) | 51.4% | |
| BMI class  (%) | Normal weight | 24.7% |
|  | Overweight | 47.1% |
|  | Obesity | 28.2% |
| Ethnic background  (%) | Pakistani | 15% |
|  | Indian | 26% |
|  | Bangladeshi | 6.8% |
|  | Somali | 15.8% |
|  | Arab | 10.3% |
|  | Other | 12.3% |
|  | Unknown | 13.7% |
| Marital status  (%) | Single | 22.6% |
|  | Married/living with a partner | 61 % |
|  | Divorced/separated | 2.7 % |
|  | Unknown | 13.7% |
| With Chronic diseases  (%) | Diabetes | 13% |
|  | Hypertension | 18.5% |
|  | Cardiovascular diseases | 5.5% |
| Education  (%) | No formal qualification | 10.3% |
|  | Secondary school or equivalent | 19.9% |
|  | Higher education: College/HNC/HND | 16.4% |
|  | Vocational qualification | 3.4% |
|  | Bachelor’s degree | 23.3% |
|  | Postgraduate degree | 13% |
|  | Unknown | 13.7% |
| Smoking  (%) | Never | 74.7% |
|  | Stopped | 9.6% |
|  | Occasionally | 3.4% |
|  | Yes, most or all days | 2.7% |
