# Supplementary material for: The impact of Ramadan intermittent fasting on anthropometric measurements and body composition: Evidence from LORANS study and a meta-analysis
Source: Front Nutr. 2023 Jan 17;10:1082217. doi: 10.3389/fnut.2023.1082217 (PMC9886683; doi:10.3389/fnut.2023.1082217)
Supplement: Supplementary material 1 — Characteristics of individuals who did not attend the second visit after Ramadan compared to LORANS participants. [file Data_Sheet_1.zip › SM6.docx]

**Supplementary Material 6**: Effect of adjustments on mean difference in anthropometric and body composition parameters from LORANS.

| **Parameter (unit)** | **Before Ramadan**  **(mean±SD)** | **After Ramadan**  **(mean±SD)** | **Base model** | **Model 2** | **Model 3** | **Model 4** | **Model 5** |
| --- | --- | --- | --- | --- | --- | --- | --- |
| Weight (kg) | 76.6±13.5 | 74.6±12.2 | -1.61 (-1.96 to -1.25)** | -1.76 (-2.19 to -1.32)** | -1.6.28)** | -0.35 (-0.81 to 0.20) | -1. |
| BMI (kg/m2) | 28.4±4.8 | 27.6±4.3 | -0.60 (-0.74 to -0.46)** | -0.67 (-0.84 to -0.50)** | -0.63 (-0.76 to -0.49)** |  | -0.46 (-0.63 to -0.27)** |
| WC (cm) | 95.8±13 | 93.8±12 | -1.95 (-3.22 to -0.69)* | -1.49 (-3.15 to 0.14) | -2.07 (-3.42 to -0.74)* |  | -1.05 (-2.50 to 0.52) |
| HC (cm) | 106.5±9.6 | 102.3±8.7 | -2.86 (-3.79 to -1.94)** | -3.03 (-4.13 to -1.92)** | -3.01 (-4.01 to -2.04)** |  | -1.83 (-2.87 to -0.70)** |
| WHR (ratio) | 0.89±0.1 | 0.91±0.1 | 0.006 (-0.005 to 0.018) | 0.011 (-0.029 to 0.026) | 0.006 (-0.005 to 0.018) | 0.011 (0.001 to 0.023) | 0.009(-0.003 to 0.021) |
| Fat mass (kg) | 23.4±10.6 | 22.5±10.1 | -1.24 (-1.70 to -0.77)** | -1.30 (-1.98 to -0.62)* | -1.19 ( -1.64 to -0.75)** | NA | -1.14 (-1.70 to -0.49)** |
| Fat (%) | 30.6±11 | 30.1±10.9 | -1.05 (-1.64 to -0.44)** | -1.04 (-1.92 to -0. 16)* | -0.97 (-1.52 to -0.43)** |  | -1.04 (-1.70 to -0.28)* |
| FFM (kg) | 51.6±10.7 | 50.7±10.2 | -0.21 (-0.68 to 0.25) | -0.24 (-0.91 to 0.43) | -0.39 (-0.94 to 0.18) |  |  |
| TBW (litre) | 37.8±7.9 | 36.1±7 | -1.45 (-1.87 to -1.04)** | -1.70 (-2.23 to -1.17)** | -1.67 (-2.16 to -1.18** | -1.45 (-1.87 to -1.01)** | NA |
| extremities PMM (kg) | 5.5±1.3 | 5.5±1.4 | 0.04 (-0.05 to 0.13) | 0.06 (-0.06 to 0.17) | NA | 0.04 (-0.05 to 0.13) |  |
| BMR (Kcal) | 1553±291 | 1527±277 | -12.8 (-23.72 to -1.84)* | -14.70 (-29.97 to 0.52) | -21.18 54)* | -24.96 (-32 to -17) ** |  |
| Trunk fat percentage (%) | 30.7±10.5 | 30.5±11.6 | -0.85 (-1.83 to 0.12) | -0.73 (-2.11 to 0.63) | -0.79 (-1.74 to 0.13) |  | -0.85 (-1.83 to 0.13) |
| Trunk FM (kg) | 13±5.7 | 12.6±6 | -0.53 (-1.07 to 0.01) | -0.57 (-1.26 to 0.12) | -0.44(-0.92 to 0.01) |  | -0.11 (-0.73 to 0.53) |
| Trunk FFM (kg) | 28.3±5 | 27.7±4.6 | -0.48 (-0.88 to -0.08)* | -0.59 (-1.15 to -0.04)* |  | -0.48 (-0.89 to 0.06) |  |
| Trunk PMM (kg) | 27.1±5 | 26.2±4.6 | -0.68 (-1.07 to -0.30)** | -0.81 (-1.34 to -0.28)* |  | -0.69 (-1.08 to -0.28)* |  |

* p-value < 0.05

** p-value <0.001

Base Model adjusts for age, sex, site, number of fasting days & day of the second measurement

Model 2 adjusts for Base model variables & energy intake

Model 3 adjusts for Base model variables & extremities PMM

Model 4 adjusts for Base model variables & FM

Model 5 adjusts for Base model variables & TBW

Note: model 2 has missing data

Black cells mean the outcome is in high correlation with the covariate
