# Supplementary material for: The impact of Ramadan intermittent fasting on anthropometric measurements and body composition: Evidence from LORANS study and a meta-analysis
Source: Front Nutr. 2023 Jan 17;10:1082217. doi: 10.3389/fnut.2023.1082217 (PMC9886683; doi:10.3389/fnut.2023.1082217)
Supplement: Supplementary material 1 — Characteristics of individuals who did not attend the second visit after Ramadan compared to LORANS participants. [file Data_Sheet_1.zip › SM7.docx]

**Supplementary Material 7**: Effect of RIF on anthropometric and body composition parameters on LORANS participants grouped by baseline BMI classes.

| **Parameter (unit)** | **BMI Class** | **Mean difference** | **p.value** |
| --- | --- | --- | --- |
|  | Normal | -2.30 | <0.001 |
| Weight (kg) | Overweight | -1.46 | 0.004 |
|  | Obesity | -1.30 | 0.148 |
|  |  |  |  |
| BMI (kg/m2) | Normal | -0.92 | <0.001 |
|  | Overweight | -0.54 | 0.002 |
|  | Obesity | -0.57 | 0.139 |
|  |  |  |  |
|  | Normal | -1.01 | 0.500 |
| WC (cm) | Overweight | -2.23 | 0.089 |
|  | Obesity | -1.24 | 0.292 |
|  |  |  |  |
|  | Normal | 0.04 | 0.982 |
| HC (cm) | Overweight | -5.01 | <0.001 |
|  | Obesity | -3.60 | 0.007 |
|  |  |  |  |
|  | Normal | 0.01 | 0.285 |
| WHR (ratio) | Overweight | 0.02 | 0.042 |
|  | Obesity | 0.02 | 0.195 |
|  |  |  |  |
|  | Normal | -0.92 | 0.728 |
| Fat mass (kg) | Overweight | -1.19 | 0.009 |
|  | Obesity | -0.16 | 0. 889 |
|  |  |  |  |
|  | Normal | -1.67 | 0.239 |
| Fat (%) | Overweight | -0.94 | 0.072 |
|  | Obesity | -0.83 | 0.600 |
|  |  |  |  |
|  | Normal | 0.04 | 0.969 |
| FFM (kg) | Overweight | -0.29 | 0.436 |
|  | Obesity | -1.20 | 0.315 |
|  |  |  |  |
|  | Normal | -0.32 | 0.811 |
| TBW (litre) | Overweight | -1.84 | <0.001 |
|  | Obesity | -1.71 | 0.039 |
|  |  |  |  |
|  | Normal | 0.42 | 0.111 |
| extremities PMM (kg) | Overweight | -0.11 | 0.212 |
|  | Obesity | -0.06 | 0.618 |
|  |  |  |  |
|  | Normal | -11.47 | 0.698 |
| BMR (Kcal) | Overweight | -11.29 | 0.259 |
|  | Obesity | -31.8 | 0.350 |
|  |  |  |  |
|  | Normal | -3.89 | 0.016 |
| Trunk fat (%) | Overweight | -0.78 | 0.352 |
|  | Obesity | 0.23 | 0.913 |
|  |  |  |  |
|  | Normal | -1.38 | 0.289 |
| Trunk FM (kg) | Overweight | -0.11 | 0.850 |
|  | Obesity | 0.58 | 0.607 |
|  |  |  |  |
|  | Normal | 0.22 | 0.782 |
| Trunk FFM (kg) | Overweight | -0.23 | 0.541 |
|  | Obesity | -1.03 | 0.148 |
|  |  |  |  |
|  | Normal | -0.02 | 0.981 |
| Trunk PMM (kg) | Overweight | -0.45 | 0.218 |
|  | Obesity | -1.22 | 0.072 |

adjusted for age, sex, site, number of fasting days & day of the second measurement
