# Supplementary material for: The impact of Ramadan intermittent fasting on anthropometric measurements and body composition: Evidence from LORANS study and a meta-analysis
Source: Front Nutr. 2023 Jan 17;10:1082217. doi: 10.3389/fnut.2023.1082217 (PMC9886683; doi:10.3389/fnut.2023.1082217)
Supplement: Supplementary material 1 — Characteristics of individuals who did not attend the second visit after Ramadan compared to LORANS participants. [file Data_Sheet_1.zip › SM8.docx]

**Supplementary Material 8:** The included studies in the meta-analysis with measured parameters and time-point of measurement.

| **First author(s) & year** | **2nd/3rd week of Ramadan** | **4th week of Ramadan** | **immediately after Ramadan** | **long after Ramadan** |
| --- | --- | --- | --- | --- |
| **Adanan 2020 (1)** |  | **BMI**  **FM**  **FP**  **WC** |  | **BMI**  **FM**  **FP**  **WC** |
|  |  |  |  |  |
|  |  |  |  |  |
|  |  |  |  |  |
| **Adnan 2014 (2)** |  | **weight** |  |  |
| **Ahmadinejad 2006 (3)** |  | **BMI**  **weight** |  |  |
|  |  |  |  |  |
| **Al Awadi 2020 (4)** |  |  |  | **Weight**  **WC** |
|  |  |  |  |  |
| **Aliasghari 2017 (5)** |  |  | **BMI**  **weight**  **WC**  **HC** |  |
|  |  |  |  |  |
|  |  |  |  |  |
|  |  |  |  |  |
| **Akanji 2000(6)** |  |  | **weight** |  |
| **Alzoughool 2019 (7)** |  | **BMI**  **Weight**  **WC** |  |  |
|  |  |  |  |  |
|  |  |  |  |  |
| **Bahmani 2013 (8)** |  |  | **BMI**  **Weight** |  |
|  |  |  |  |  |
| **Bashier 2018 (9)** |  |  |  | **Weight** |
| **Bernieh 2010 (10)** |  | **Weight** |  |  |
| **Bencharif 2017 (11)** | **BMI**  **weight**  **FP**  **WC** |  |  | **BMI**  **weight**  **FP**  **WC** |
|  |  |  |  |  |
|  |  |  |  |  |
|  |  |  |  |  |
| **Bouida 2017 (12)** |  | **BMI**  **weight** |  | **BMI**  **weight** |
|  |  |  |  |  |
| **Dasgupta 2017 (13)** |  |  | **BMI**  **weight**  **WC** |  |
|  |  |  |  |  |
|  |  |  |  |  |
| **Devendra 2009 (14)** |  |  | **weight** |  |
| **Ebrahimi 2018 (15)** |  |  | **BMI**  **weight**  **FP**  **WC**  **HC** |  |
|  |  |  |  |  |
|  |  |  |  |  |
|  |  |  |  |  |
|  |  |  |  |  |
| **Elfert 2011 (16)** |  |  | **BMI** |  |
| **Faris 2019 (17)** |  | **BMI**  **weight**  **FM**  **WC**  **HC**  **TBW**  **MM** |  |  |
|  |  |  |  |  |
|  |  |  |  |  |
|  |  |  |  |  |
|  |  |  |  |  |
|  |  |  |  |  |
|  |  |  |  |  |
| **Faris 2012 (18)** | **BMI**  **weight**  **FP**  **WC**  **HC**  **WHR** |  |  | **BMI**  **weight**  **FP**  **WC**  **HC**  **WHR** |
|  |  |  |  |  |
|  |  |  |  |  |
|  |  |  |  |  |
|  |  |  |  |  |
|  |  |  |  |  |
| **Feizollahzadeh 2014 (19)** |  |  | **BMI**  **weight** |  |
|  |  |  |  |  |
| **Finch 1998 (20)** |  | **weight** |  | **weight** |
| **Gholami 2019 (21)** |  |  | **BMI**  **WC** |  |
|  |  |  |  |  |
| **Hassanein 2019 (22)** |  |  |  | **weight**  **WC** |
|  |  |  |  |  |
| **Hassanein 2011 (23)** |  |  |  | **weight** |
| **Khan 2017 (24)** |  | **BMI**  **weight**  **WC**  **HC**  **WHR** |  | **BMI**  **weight**  **WC**  **HC**  **WHR** |
|  |  |  |  |  |
|  |  |  |  |  |
|  |  |  |  |  |
|  |  |  |  |  |
| **Khattak 2013 (25)** | **BMI**  **weight**  **WHR** |  |  |  |
|  |  |  |  |  |
|  |  |  |  |  |
| **Karatoprak 2013 (26)** |  |  | **weight** |  |
| **Kiyani 2015 (27)** |  |  | **weight** |  |
| **Muhammad 2018 (28)** |  | **BMI**  **weight**  **FP**  **WC** | **BMI**  **weight**  **FP**  **WC** |  |
|  |  |  |  |  |
|  |  |  |  |  |
|  |  |  |  |  |
| **Madkour 2019 (29)** |  | **BMI**  **weight**  **FM**  **FP**  **MM**  **WC**  **HC**  **TBW**  **WHR** |  |  |
|  |  |  |  |  |
|  |  |  |  |  |
|  |  |  |  |  |
|  |  |  |  |  |
|  |  |  |  |  |
|  |  |  |  |  |
|  |  |  |  |  |
|  |  |  |  |  |
| **Malekmakan 2017 (30)** |  |  | **BMI**  **weight**  **WC**  **HC** |  |
|  |  |  |  |  |
|  |  |  |  |  |
|  |  |  |  |  |
| **Khaled 2009 (31)** | **BMI**  **weight**  **WC**  **WHR** |  |  | **BMI**  **weight**  **WC**  **WHR** |
|  |  |  |  |  |
|  |  |  |  |  |
|  |  |  |  |  |
| **Nachvak 2018 (32)** |  | **BMI**  **weight**  **FM**  **FP**  **WHR** |  | **BMI**  **weight**  **FM**  **FP**  **WHR** |
|  |  |  |  |  |
|  |  |  |  |  |
|  |  |  |  |  |
|  |  |  |  |  |
| **Namaghi 2019 (33)** |  |  | **BMI**  **weight**  **FM**  **MM**  **TBW** |  |
|  |  |  |  |  |
|  |  |  |  |  |
|  |  |  |  |  |
|  |  |  |  |  |
| **Nematy 2012 (34)** |  |  | **BMI**  **weight**  **WC** |  |
|  |  |  |  |  |
|  |  |  |  |  |
| **Norouzy 2013 (35)** |  |  | **BMI**  **weight**  **FM**  **FP**  **WC**  **HC** |  |
|  |  |  |  |  |
|  |  |  |  |  |
|  |  |  |  |  |
|  |  |  |  |  |
|  |  |  |  |  |
| **Norouzy 2012 (36)** |  |  | **BMI**  **weight**  **WC**  **HC** |  |
|  |  |  |  |  |
|  |  |  |  |  |
|  |  |  |  |  |
| **Ongsara 2017 (37)** |  | **BMI**  **weight**  **FM**  **FP**  **WC**  **MM** |  | **BMI**  **weight**  **FM**  **FP**  **WC**  **MM** |
|  |  |  |  |  |
|  |  |  |  |  |
|  |  |  |  |  |
|  |  |  |  |  |
|  |  |  |  |  |
| **Patel 2007 (38)** |  |  | **BMI**  **weight** |  |
|  |  |  |  |  |
| **Sahin 2013 (39)** |  |  | **BMI**  **weight**  **WC** |  |
|  |  |  |  |  |
|  |  |  |  |  |
| **Shariatpanahi 2008 (40)** |  |  | **BMI**  **weight**  **WC** |  |
|  |  |  |  |  |
|  |  |  |  |  |
| **Shehab 2012 (41)** |  | **BMI**  **weight**  **WC** |  | **BMI**  **weight**  **WC** |
|  |  |  |  |  |
|  |  |  |  |  |
| **Sulu 2010 (42)** |  | **BMI** |  |  |
| **Syam 2016 (43)** |  | **BMI**  **weight**  **FM**  **TBW** |  |  |
|  |  |  |  |  |
|  |  |  |  |  |
|  |  |  |  |  |
| **Ghania 2015(44)** | **BMI**  **weight**  **WC** |  |  |  |
|  |  |  |  |  |
|  |  |  |  |  |
| **Toony 2018 (45)** | **BMI**  **weight**  **WC** |  |  |  |
|  |  |  |  |  |
|  |  |  |  |  |
| **Yarahmadi 2003 (46)** | **BMI**  **WHR** | **BMI**  **WHR** |  |  |
|  |  |  |  |  |
| **Shariatpanahi 2012 (47)** |  |  | **BMI**  **WC** |  |
|  |  |  |  |  |
| **Khan 2012 (24)** |  | **weight**  **WC**  **HC**  **WHR** |  |  |
|  |  |  |  |  |
|  |  |  |  |  |
|  |  |  |  |  |
| **Imtiaz 2016 (48)** |  | **weight** |  |  |
| **Laajam 1990 (49)** |  |  | **weight** |  |
| **Pathan and Patil 2010 (50)** |  |  | **weight** |  |
| **Traore, et al. 2014 (51)** |  | **weight** |  | **weight** |
| **Abdullah et al 2020 (52)** |  | **BMI**  **WC** |  |  |
|  |  |  |  |  |
| **Al-Rawi et al 2020 (53)** |  |  | **weight**  **BMI**  **WC**  **HC**  **TBW**  **FP**  **FM**  **FFM**  **MM** |  |
|  |  |  |  |  |
|  |  |  |  |  |
|  |  |  |  |  |
|  |  |  |  |  |
|  |  |  |  |  |
|  |  |  |  |  |
|  |  |  |  |  |
|  |  |  |  |  |
| **Das et al 2021 (54)** |  |  |  | **weight**  **BMI** |
|  |  |  |  |  |
| **Farag et al 2020 (55)** |  |  | **weight**  **BMI**  **WC** |  |
|  |  |  |  |  |
|  |  |  |  |  |
| **Gad et al 2022 (56)** |  |  | **BMI** |  |
| **Harbuwono et al 2021 (57)** |  |  |  | **weight**  **BMI** |
|  |  |  |  |  |
| **Ismail et al 2021 (58)** |  |  |  | **BMI** |
| **Jahrami et al 2021 (59)** |  |  | **weight**  **BMI**  **WC**  **HC**  **WHR**  **FP**  **FM** |  |
|  |  |  |  |  |
|  |  |  |  |  |
|  |  |  |  |  |
|  |  |  |  |  |
|  |  |  |  |  |
|  |  |  |  |  |
| **López-Bueno et al 2021 (60)** |  | **weight**  **BMI**  **WC**  **HC**  **TBW**  **FP**  **FM**  **WHR**  **MM** |  |  |
|  |  |  |  |  |
|  |  |  |  |  |
|  |  |  |  |  |
|  |  |  |  |  |
|  |  |  |  |  |
|  |  |  |  |  |
|  |  |  |  |  |
|  |  |  |  |  |
| **Mohamed et al 2021 (61)** |  |  |  | **BMI** |
| **Mohammadzadeh et al 2021 (62)** |  |  | **BMI** |  |
| **Urooj et al 2020 (63)** |  |  | **weight**  **BMI**  **FP**  **WHR**  **MM** |  |
|  |  |  |  |  |
|  |  |  |  |  |
|  |  |  |  |  |
|  |  |  |  |  |
| **Yazdanyar et al 2020 (64)** |  |  | **weight**  **BMI**  **WC**  **WHR** |  |
|  |  |  |  |  |
|  |  |  |  |  |
|  |  |  |  |  |
| **LORANS 2019** |  |  | **BMI**  **weight**  **FM**  **FP**  **WC**  **HC**  **TBW** |  |
|  |  |  |  |  |
|  |  |  |  |  |
|  |  |  |  |  |
|  |  |  |  |  |
|  |  |  |  |  |
|  |  |  |  |  |
