# Supplementary figures and images for: The impact of Ramadan intermittent fasting on anthropometric measurements and body composition: Evidence from LORANS study and a meta-analysis
Source: Front Nutr. 2023 Jan 17;10:1082217. doi: 10.3389/fnut.2023.1082217 (PMC9886683; doi:10.3389/fnut.2023.1082217)

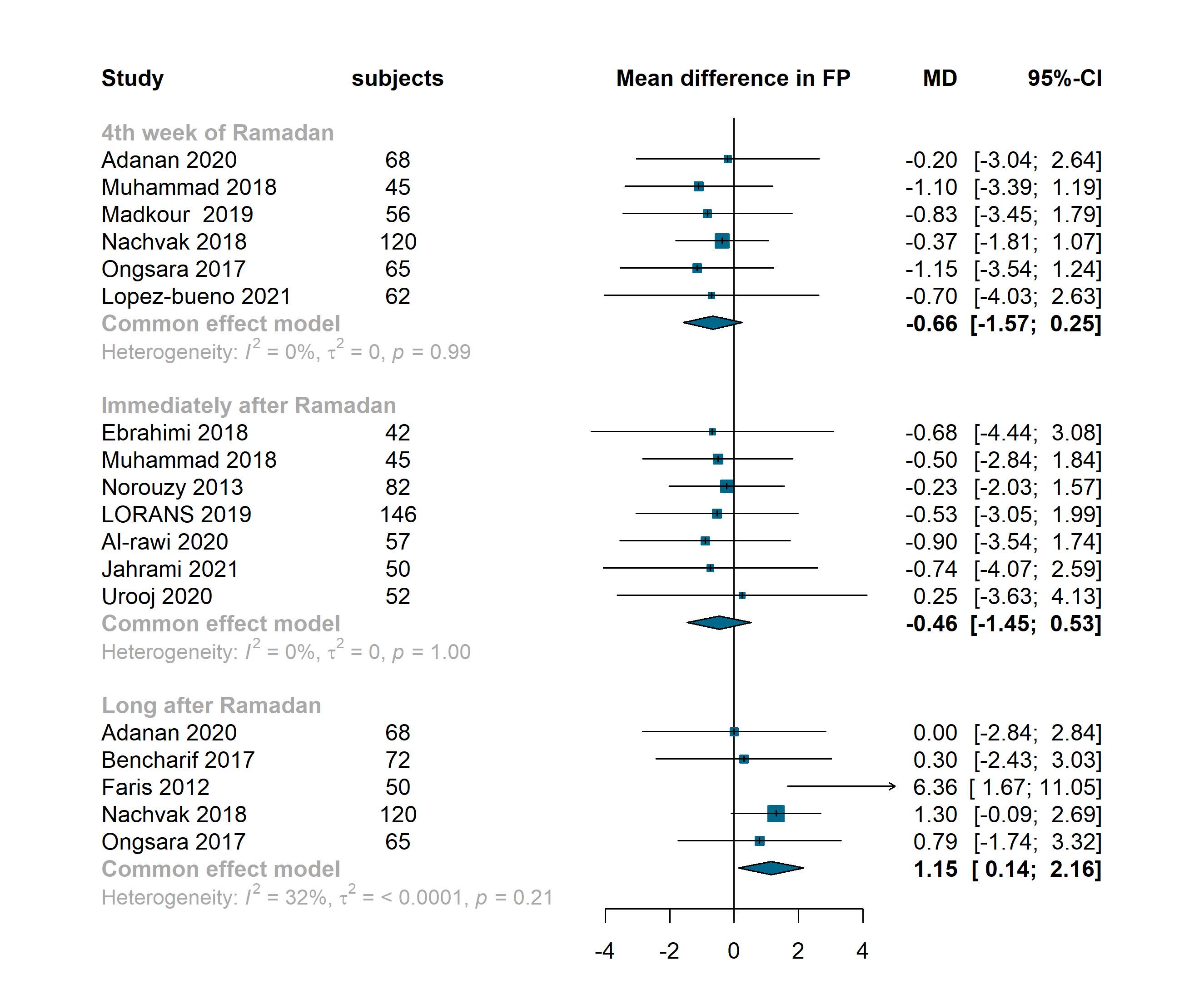

Supplement: Supplementary material 9 — Fixed effect meta-analysis of RIF effect on fat percentage. [file Image_1.JPEG]

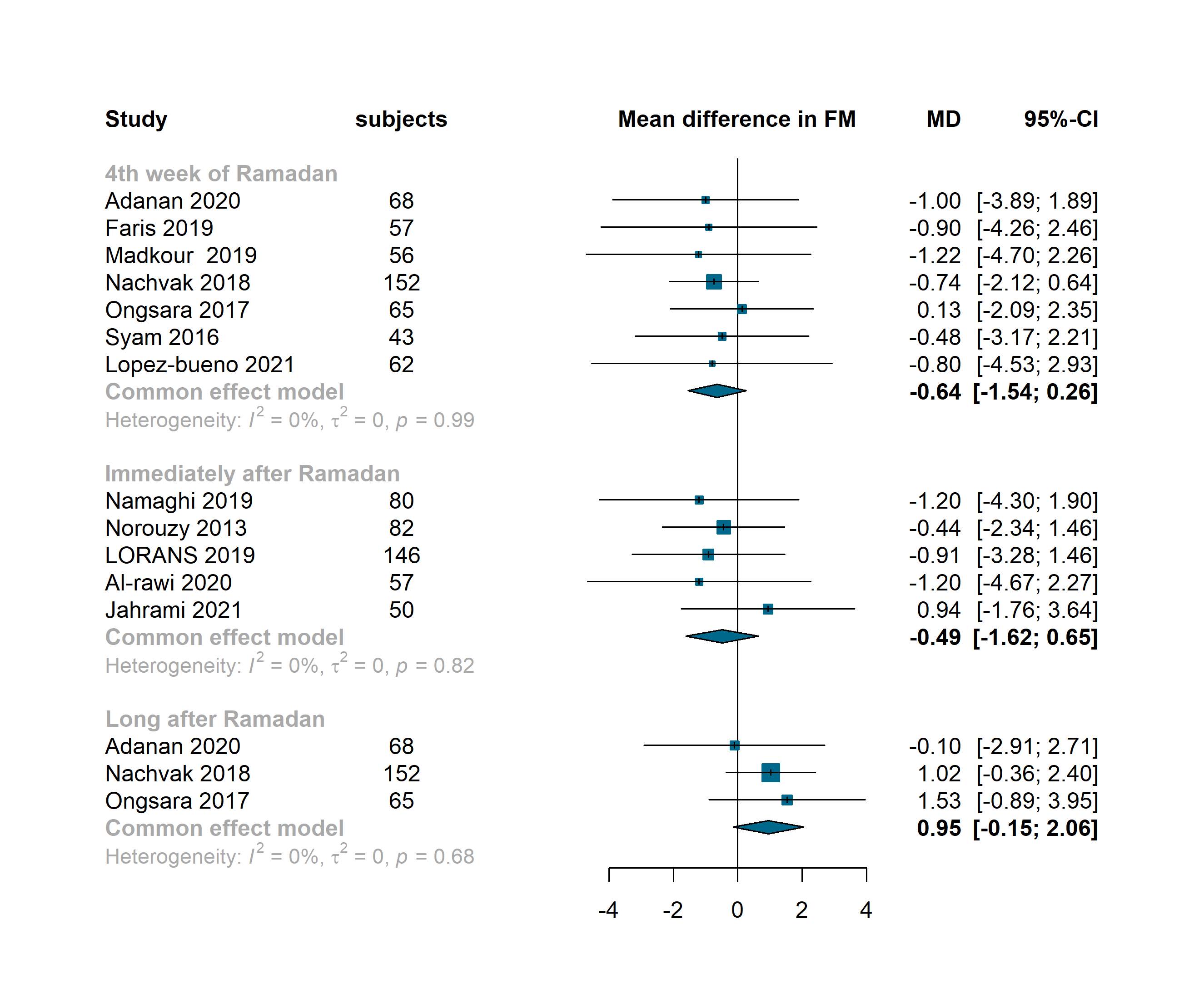

Supplement: Supplementary material 10 — Fixed effect meta-analysis of RIF effect on fat mass. [file Image_2.JPEG]

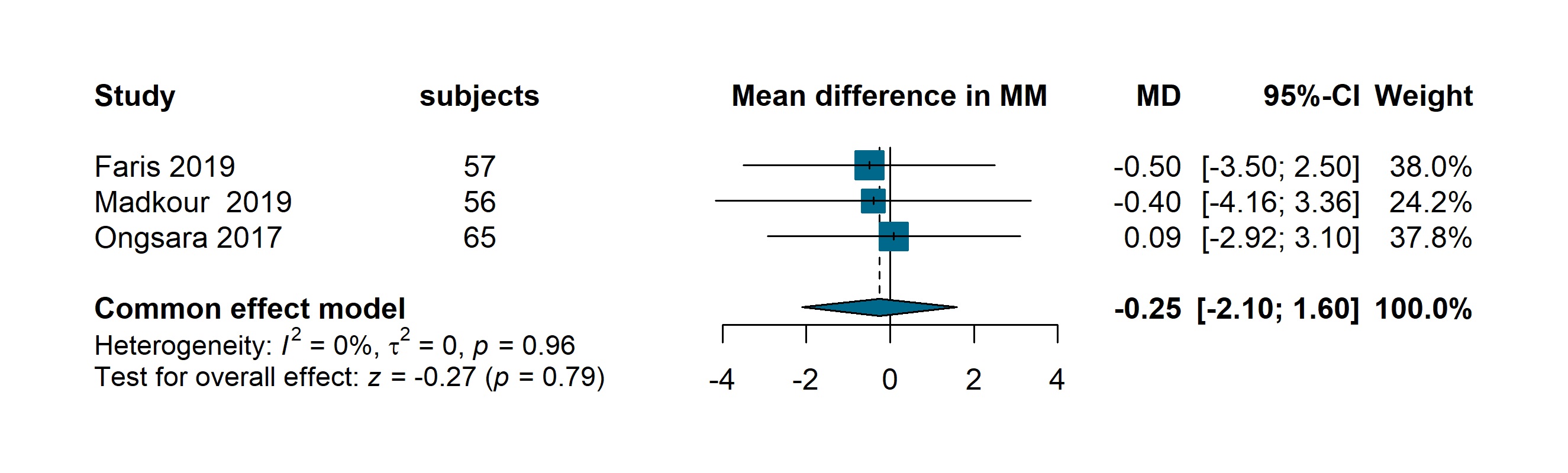

Supplement: Supplementary material 11 — Fixed effect meta-analysis of RIF effect on muscle mass during the fourth week of Ramadan. [file Image_3.JPEG]

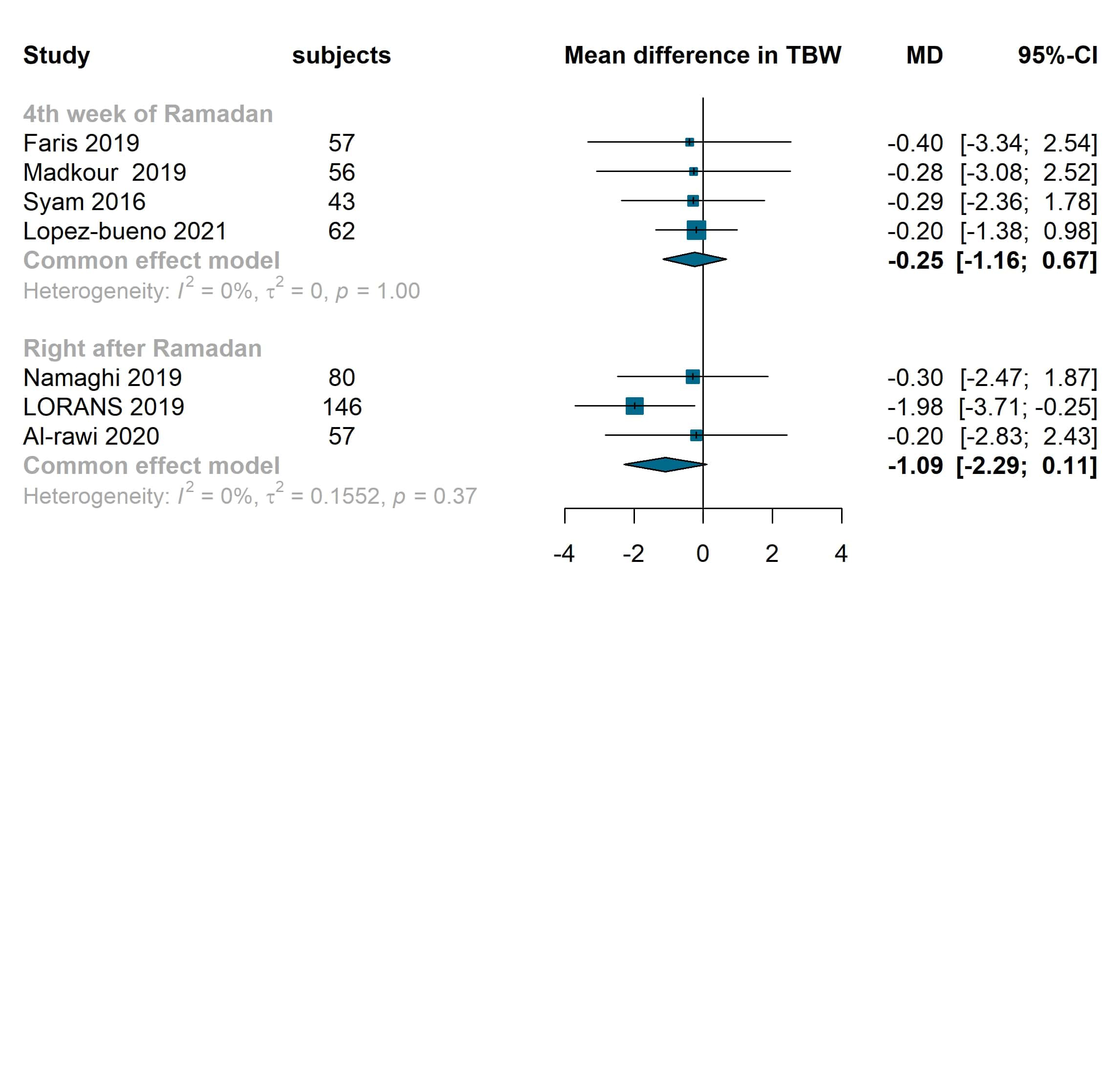

Supplement: Supplementary material 12 — Fixed effect meta-analysis of RIF effect on total body water. [file Image_4.JPEG]

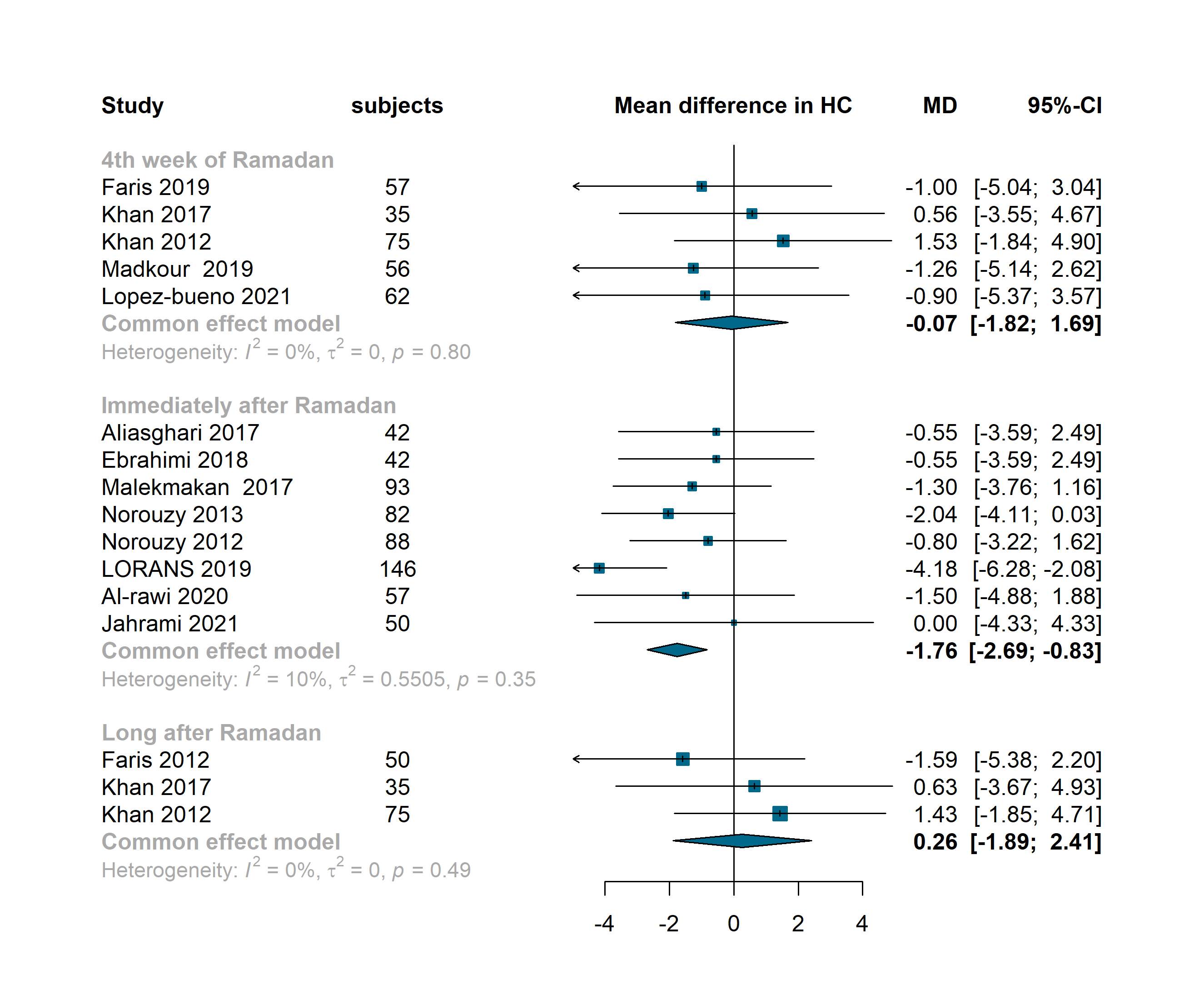

Supplement: Supplementary material 13 — Fixed effect meta-analysis of RIF effect on hip circumference. [file Image_5.JPEG]

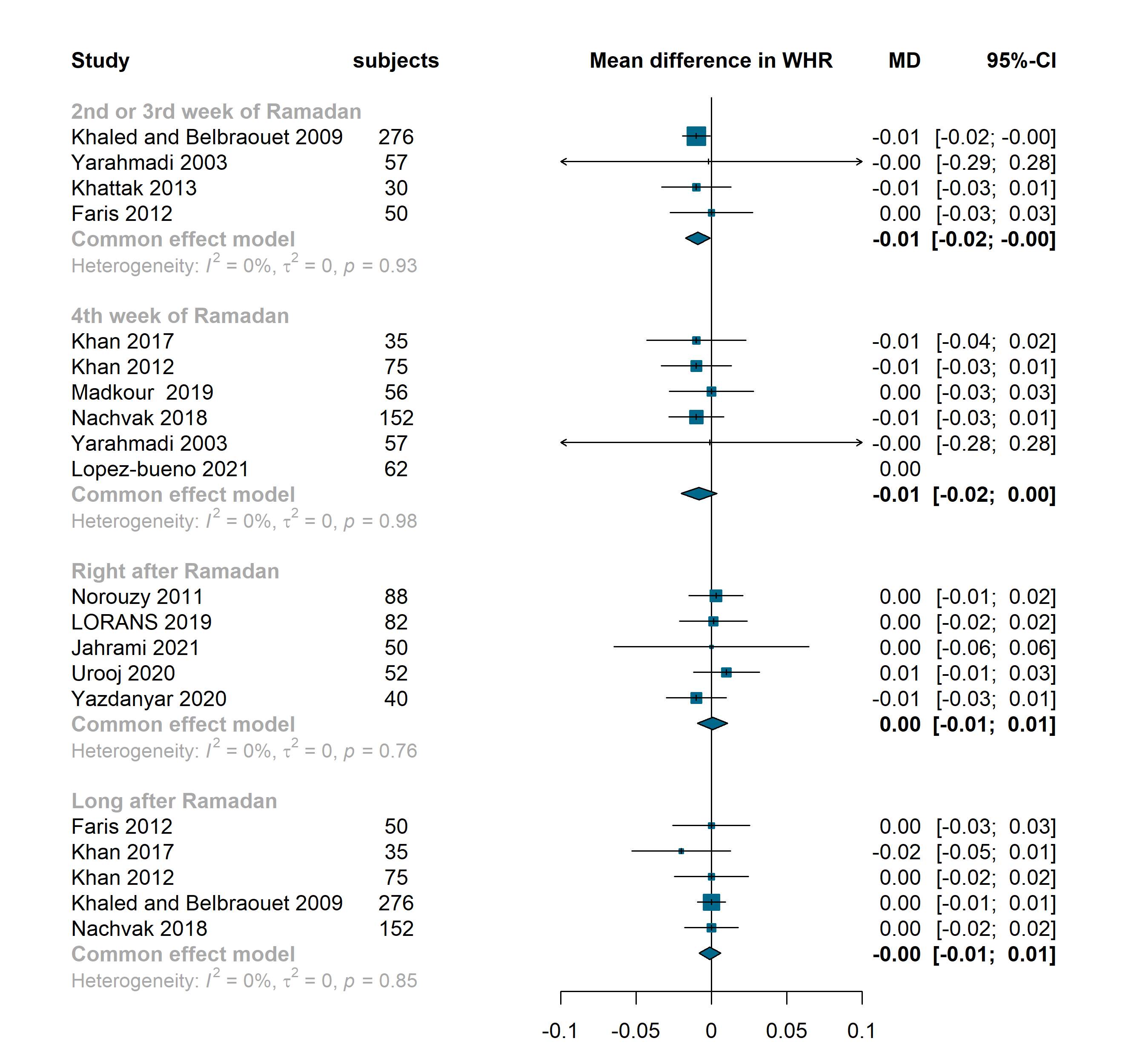

Supplement: Supplementary material 14 — Fixed effect meta-analysis of RIF effect on the waist to hip ratio. [file Image_6.JPEG]
